# Substrate-dependent oxidative inactivation of a W-dependent formate dehydrogenase involving selenocysteine displacement†

**DOI:** 10.1039/d4sc02394c

**Published:** 2024-07-16

**Authors:** Guilherme Vilela-Alves, Rita R. Manuel, Aldino Viegas, Philippe Carpentier, Frédéric Biaso, Bruno Guigliarelli, Inês A. C. Pereira, Maria João Romão, Cristiano Mota

**Affiliations:** a Associate Laboratory i4HB – Institute for Health and Bioeconomy, NOVA School of Science and Technology, Universidade NOVA de Lisboa 2829-516 Caparica Portugal cd.mota@fct.unl.pt mjr@fct.unl.pt; b UCIBIO, Applied Molecular Biosciences Unit, Department of Chemistry, NOVA School of Science and Technology, Universidade NOVA de Lisboa 2829-516 Caparica Portugal; c Instituto de Tecnologia Química e Biológica António Xavier, Universidade Nova de Lisboa Av. da República 2780-157 Oeiras Portugal ipereira@itqb.unl.pt; d European Synchrotron Radiation Facility Grenoble France; e Institut de Recherche Interdisciplinaire de Grenoble (IRIG), Laboratoire Chimie et Biologie des Métaux (LCBM), Université Grenoble Alpes, CNRS, CEA Grenoble France; f Aix Marseille Univ, CNRS, BIP, Laboratoire de Bioénergétique et Ingénierie des Protéines Marseille 13402 France

## Abstract

Metal-dependent formate dehydrogenases are very promising targets for enzyme optimization and design of bio-inspired catalysts for CO_2_ reduction, towards innovative strategies for climate change mitigation. For effective application of these enzymes, the catalytic mechanism must be better understood, and the molecular determinants clarified. Despite numerous studies, several doubts persist, namely regarding the role played by the possible dissociation of the SeCys ligand from the Mo/W active site. Additionally, the oxygen sensitivity of these enzymes must also be understood as it poses an important obstacle for biotechnological applications. This work presents a combined biochemical, spectroscopic, and structural characterization of *Desulfovibrio vulgaris* FdhAB (*Dv*FdhAB) when exposed to oxygen in the presence of a substrate (formate or CO_2_). This study reveals that O_2_ inactivation is promoted by the presence of either substrate and involves forming a different species in the active site, captured in the crystal structures, where the SeCys ligand is displaced from tungsten coordination and replaced by a dioxygen or peroxide molecule. This form was reproducibly obtained and supports the conclusion that, although W-*Dv*FdhAB can catalyse the oxidation of formate in the presence of oxygen for some minutes, it gets irreversibly inactivated after prolonged O_2_ exposure in the presence of either substrate.

## Abstract 2

Our study shows, at near-atomic resolution, how W-*Dv*FdhAB inactivates over time when exposed to either of its substrates and O_2_.

## Graphical abstract

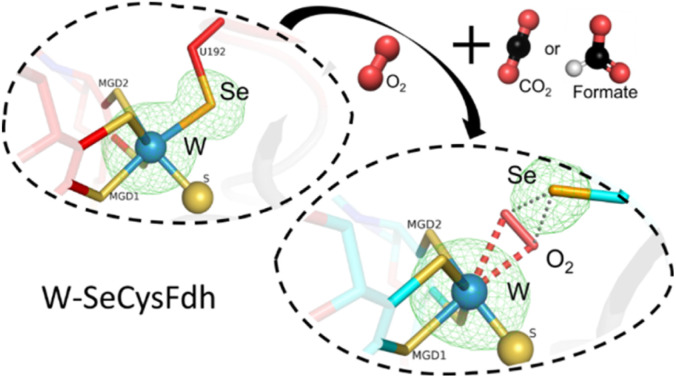

## Introduction

While there is an urgent need for climate change mitigation measures, conversion of atmospheric CO_2_ into added value products entails particularly challenging chemical reactions, due to the inherent stability of this molecule.^1–4^ In this context, the highly active, efficient, and selective metal-dependent Formate Dehydrogenases (Fdh) are very appealing systems that enable harnessing millions of years of natural evolution.^5,6^ As such, there is an increasing interest in developing and deploying these natural enzymes and optimized versions, as well as in creating bio-inspired catalytically active model compounds.^7–12^

Metal-dependent Fdhs harbour at the catalytic site a Mo/W ion coordinating a (seleno)cysteine (SeCys/Cys), four sulfur atoms from two dithiolenes of two Molybdopterin Guanine Dinucleotides (MGD) and one terminal sulfido ligand (–SH/=S). Additionally, [4Fe–4S] clusters are present to convey electrons to/from the physiological partners. While much is already known regarding these enzymes, their catalytic mechanism remains unclear, despite several hypotheses proposed so far. Among these, major divergencies persist, namely whether SeCys has to dissociate from the Mo/W ion during catalysis to allow substrate binding to the W ion^13,14^ or if, instead, a direct hydride transfer occurs between the substrate and the sulfido ligand, with the substrate located in the second coordination sphere.^15^ To date, computational work has addressed several mechanistic hypotheses,^16–19^ but without a clear answer.

Most metal-dependent Fdhs are known to be O_2_-sensitive, easily losing their catalytic activity upon oxygen exposure,^20^ which constitutes a drawback for biotechnological applications. This was addressed for the first time in 1939 by Gale^21^ who described that *Bacterium coli* (now *E. coli*) extracts containing formate dehydrogenase activity react with O_2_ in two different ways, either indirectly (likely occurring at the FeS clusters, and using O_2_ as an electron acceptor) or directly (likely at the W centre), suggesting that this may result in enzyme inactivation. In 1975, Enoch and Lester^22^ were able to fully reproduce these results, with the isolated enzyme. Additionally, they showed that reduction of dichlorophenolindophenol, ferricyanide, or nitro blue tetrazolium by Fdh was immediately hampered by the presence of O_2_, while complete inactivation of the enzyme required a measure of time, thus restating the role of O_2_ as a competing electron acceptor for Fdh. Remarkably, Graham *et al.*^23^ recently showed that the *Desulfovibrio vulgaris* Hildenborough Fdh2 can oxidize formate in the presence of atmospheric O_2_, unlike most metal-dependent Fdhs, and can also use O_2_ as electron acceptor for formate oxidation, reducing it mostly to H_2_O_2_.

To improve the properties of Fdhs and increase their oxygen tolerance, a detailed structural knowledge of the exact nature of the damage imposed by O_2_ is paramount. This information may spearhead the development of protection strategies and/or ways to prevent or recover activity upon inactivation. Recently, it was reported that *D. vulgaris* W/SeCys-FdhAB (*Dv*FdhAB, Fdh1) is irreversibly inactivated by O_2_ in the presence of formate, after a period of time.^24,26^

To better understand the inactivation process, this work reports detailed crystallographic studies of *Dv*FdhAB under oxygen exposure along time, in the presence of formate or CO_2_, which were supported by spectroscopic and kinetic studies. These experiments enabled the structural characterization of the oxygen induced damage of *Dv*FdhAB and disclose the conditions in which it occurs.

## Results and discussion

### O_2_-inactivation of formate-reduced *Dv*FdhAB involves SeCys192 displacement

Recently, it was reported a time-resolved crystallography study of the structural changes of *Dv*FdhAB occurring upon formate reduction in anoxic conditions.^25^ A similar approach was employed to gain further insight into the structural changes occurring when the formate-reduced enzyme is exposed to O_2_, thus rendering it inactivated.^24,26^ Since the formate-reduced enzyme is more sensitive to O_2_, formate-treated *Dv*FdhAB crystals were first prepared in the anaerobic chamber (<0.1 ppm of O_2_). In a previous work,^26^ it was shown that an allosteric disulfide bond (C845–C872) controls the activity of *Dv*FdhAB, with the enzyme being activated by its reduction. Thus, in the current work, the non-activated enzyme (C845–C872 disulfide bond oxidized) was used to slow down the catalytic reaction,^25^ aiming at trapping putative intermediates. The formate-reduced *Dv*FdhAB crystals were flash cooled in liquid nitrogen at different time points after exposure to atmospheric O_2_ (Tables 1 and S1†). One of the crystals was cooled before O_2_ exposure (Control_Red) to confirm that the protein was fully reduced at the beginning of the experiment, and in agreement with the formate-reduced structure (PBD_ID: 6SDV)^24^ (Fig. S1†).

**Table 1:** Summary of the different CO_2_ and/or oxygen soaking experiments performed with *Dv*FdhAB in this and other works

Structure/resolution	Anaerobic co-crystallization with formate	Exposed to O_2_	Transferred to a new oxygenated drop (ND)	Formate present during O_2_ exposure	Form present [ref]
As-isolated/2.10 Å	No	Yes	—	No	As-isolated^24^
PDB_ID: 6SDR					
Formate-reduced/1.90 Å	Yes	No	—	—	Reduced^24^
PDB_ID: 6SDV					
Control_Red/2.00 Å	Yes	No	—	—	Reduced [this work]
PDB_ID: 8RC8					
Reox_12 min/1.91 Å	Yes	Yes	No	Yes	As-isolated^25^
PDB_ID: 8BQL					
Reox_120 min/2.06 Å	Yes	Yes	No	Yes	W–O=O⋯SeCys [this work]
PDB_ID: 8RC9					
Reox_ND_NoFormate/1.66 Å	Yes	Yes	Yes	No	As-isolated [this work]
PDB_ID: 8RCA					
Reox_ND_Formate/2.11 Å	Yes	Yes	Yes	Yes	W–O=O⋯SeCys [this work]
PDB_ID: 8RCB					
HP_CO_2_ (high pressure CO_2_)/2.30 Å	No	Yes	No	No	W–O=O⋯SeCys [this work]
PDB_ID: 8RCC					

**Fig. 1:**
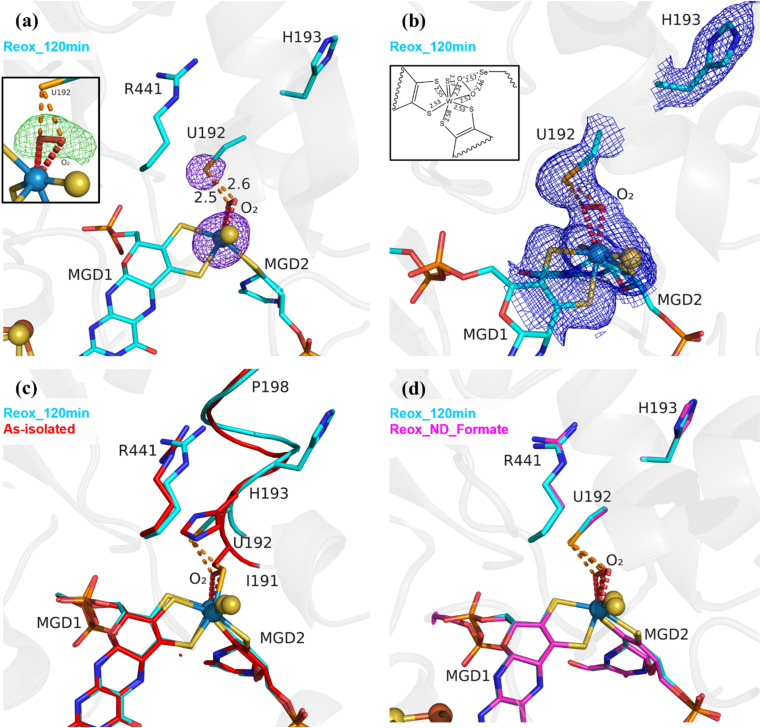
Exposure of formate-reduced *Dv*FdhAB to oxygen leads to SeCys192 displacement. (a) *Dv*FdhAB Reox_120 min structure (cyan) and corresponding anomalous electron density map contoured at 5*σ* (purple mesh). U192, H193, R441, the two MGD co-factors coordinating the W ion and the dioxygen molecule (red) are shown as sticks. In the insert, the omit difference map for the dioxygen molecule is represented, contoured at 3*σ* (green mesh). (b) Same structure as (a), with the 2Fo-Fc electron density map contoured at 1*σ* (blue mesh). In the insert, a 2D representation of the W active site indicates the relevant bond lengths (in Å). (c) Superposition of *Dv*FdhAB as-isolated (red) and Reox_120 min (cyan) structures. U192, H193, R441, the two MGD co-factors and the dioxygen molecule (red) are shown as sticks, the helix (I191–P198) is shown as ribbon. (d) Superposition of *Dv*FdhAB Reox_120 min (cyan) and Reox_ND_Formate (magenta) structures. U192, H193, R441, the two MGD co-factors and the dioxygen molecule (red) are shown as sticks.

As previously reported,^25^ (PDB_ID: 8BQL), after 12 min of O_2_ exposure (Reox_12 min) the resulting structure was superimposable with the *Dv*FdhAB as-isolated oxidized form (PDB_ID: 6SDR),^24^ without any sign of damage to the cofactors (Fig. S2†). However, after 20 minutes of O_2_ exposure, the electron density maps near the active site were difficult to interpret, suggesting a mixture of states which were later confirmed by refining with partial occupancies (Fig. S3†). After 2 h of O_2_ exposure, the modelled structure (Reox_120 min) presented extensive changes in the W active site, when compared to all published structures of *Dv*FdhAB. The most striking difference is the fact that SeCys is no longer coordinated to the W ion, with Se⋯W distance of 4.2 Å (Fig. 1a and b, Table S2†). The electron density map reveals that the coordination position, that has been left vacant by this displacement, is systematically replaced by a new ligand of diatomic shape in a *η*^2^ fashion (the respective omit map is shown in Fig. 1a) (W–O bond distances of ∼2.3 Å and ∼2.5 Å (for the highest resolution structure Reox_120 min (Table S2†)). Since O_2_ is expected to react with the active site, this new ligand was interpreted as being either a dioxygen or a peroxide molecule. Alternative modelling of different ligands was tried as shown in Fig. S4.† From these it was concluded that single heteroatom ligands are insufficient to fully account for the electron density and that a dioxygen/peroxide molecule simultaneously fits the electron density better and offers a more coherent interpretation of all the data obtained in this work. Two water molecules with 50% occupancy also acceptably fit the electron density but one of the molecules refines with a very low *B* factor (8 Å^2^) when compared with neighbouring atoms (∼20–30 Å^2^). Alternative SeCys oxidation states were also tested but could not reasonably account for the electron density of the FdhAB inactive state.

The Se⋯O distance determined from the crystal structure (∼2.5 Å) is in agreement with the computational model (see in ESI†) when SeCys is dissociated from the W ion and deprotonated (2.7 Å) (Fig. S5 and S6†). On the other hand, the calculated value for an unbound protonated SeCys system (3.6 Å, Table S2†) indicates that the Reox_120 min structure is inconsistent with SeCys protonation, assumed to be deprotonated given the expected low pKa of the selenol group (p*K*a = 5.4). In the absence of the protein environment, at pH 8.0 (pH of the crystallization buffer) SeCys is expected to be negatively charged. Thus, one can propose a putative Debye permanent dipole-induced dipole interaction between the charged selenate and the polarizable dioxygen. This state of the enzyme was systematically reproduced in multiple experiments (>4 structures). Unambiguous confirmation of the W–Se dissociation is provided by strong anomalous density peaks, detectable at an excess of 11*σ* (Reox_120 min, PDB_ID: 8RC9), and centred at the positions of the Se atom of SeCys192 and of the W atom (Fig. 1a). No trace of anomalous signal was observed at the dioxygen position. Finally, no evidence of oxidative damage to any of the four [4Fe–4S] clusters was found in the crystal structure. All atoms of the clusters were refined with full occupancy, and the *B*-factors are consistent with those of the neighbouring atoms.

A similar coordination mode of a dioxygen/peroxide molecule to a Mo centre has been reported for the *D. gigas* (*Dg*) AOR (a member of the Xanthine Oxidase family) where this molecule replaces the labile equatorial hydroxyl ligand, being bound to the Mo ion in a *η*^2^ fashion with Mo–O bond lengths of 1.96 and 2.49 Å for crystals of *Dg*AOR soaked with peroxide (PDB_ID: 4C80, 1.5 Å resolution) (Fig. S7†).^27^

In the Reox_120 min structure, the sulfido ligand and the Se atom were refined with full occupancy revealing *B*-factors higher than the W and the dithiolene ligands (Table S3†). Firstly, this observation suggests that there is an increased uncertainty on the sulfido and Se atom positions due to the reorganization of the W coordination sphere. However, it may also correspond to partial sulfido loss/replacement by an oxygen atom. This same observation is confirmed in the other two structures reported here, with displaced SeCys (W–O=O⋯Se (see below)) (Table S3†).

Along with the dissociation of SeCys192, the helix I191–P198 also changes its position with a screw-like displacement (*e.g.*, 2.9 Å between H193 Cα in as-isolated and Reox_120 min) (Fig. 1c). Particularly interesting is the large change in H193 that is now facing away from the active site and towards the formate tunnel, with the imidazole ring lodged in the binding position of a glycerol molecule (GOL2) previously reported in several *Dv*FdhAB structures (*e.g.*, PDB_ID: 6SDR) (Fig. 1 and S8a†). The twisting effect propagates through the α-helix, being attenuated as it moves away from SeCys192. At residues T196/V197 the conformational change is much smaller and comparable with the one between the as-isolated (PDB_ID: 6SDR) and reduced forms (PDB_ID: 6SDV),^24^ and at P198 the structures are essentially superimposable. The conformational changes observed in helix I191–P198 with the displacement of SeCys192 and further oxygen inactivation is reminiscent of the behaviour observed in *Clostridium beijerinckii* [FeFe] hydrogenase.^28^ The displacement of I191 also leads to conformational changes in the sidechain of the hydrophobic core: W533, F160 and F537 (not represented).^24,25^

To overcome the effect of the slow diffusion rate of O_2_ into the anaerobic drop, anaerobic crystals were transferred to a new drop (ND) with similar composition to the mother liquor containing 10 mM of sodium formate, but already oxygenated, and were further exposed to atmospheric oxygen for 34 min (Tables 1 and S1†) (a shorter exposure time led to a mixture of states, while a longer one led to poorly diffracting crystals).

The resulting structure (Reox_ND_Formate) was nearly identical to Reox_120 min (Fig. 1d), showing that the rate of oxygen diffusion plays a significant role in the rate of the SeCys dissociation process. In addition, formate-treated crystals were transferred to a new oxygenated drop without formate and exposed to atmospheric O_2_ for 1 h (Tables 1 and S1†) (Reox_ND_NoFormate). The corresponding structure showed the enzyme without any sign of oxygen-induced changes (Fig. S8†) and the structure superimposes with the as-isolated form (PDB_ID: 6SDR)^24^ with no apparent differences in the active site or [4Fe–4S] centres. This result reveals an essential role of the substrate formate in the SeCys dissociation.

### Exposure to CO_2_ and O_2_ also inactivates *Dv*FdhAB and involves SeCys192 displacement

**Fig. 2:**
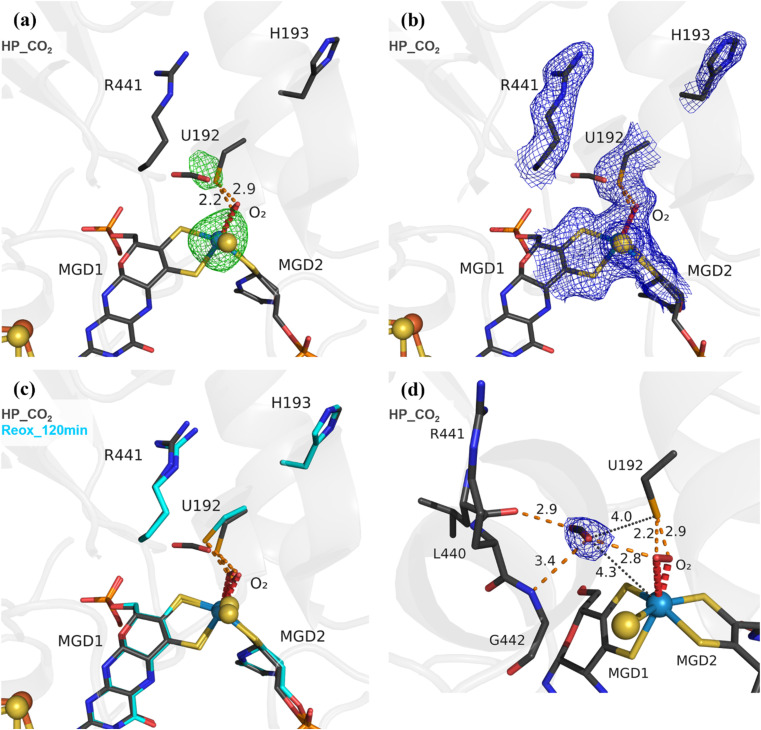
Exposure of *Dv*FdhAB to carbon dioxide and oxygen leads to SeCys192 displacement. (a) Structure of *Dv*FdhAB soaked with CO_2_ under high pressure, aerobically, HP_CO_2_ (dark grey) and respective anomalous electron density map, at 3.5*σ* (green mesh). U192, H193, R441, a CO_2_ molecule, the two MGD co-factors and the dioxygen molecule (red) coordinating the W ion are shown as sticks. (b) Same structure as in (a) with the respective electron density map (2Fo-Fc) contoured at 1*σ* (blue mesh). (c) Superposition of *Dv*FdhAB Reox_120 min (cyan) and HP_CO_2_ (dark grey) structures. U192, H193, R441, a CO_2_ molecule, the two MGD co-factors and the dioxygen molecule (red) are shown as sticks. (d) Same structure as in (a) with the CO_2_ electron density map (2Fo-Fc) contoured at 1*σ* (blue mesh). The orange dashes represent the contacts between the CO_2_ rear-facing oxygen atom and L440 carbonyl group and the forward-facing oxygen atom and both the G442 NH group and the dioxygen molecule. Additionally, the distances between the CO_2_ forward-facing oxygen atom and the W center and SeCys Se atom are shown as black dotted lines.

To study the effect of O_2_ in the presence of the CO_2_ substrate, High Pressure (HP) gas soaking experiments were performed at the HP Macromolecular Crystallography Lab (HPMX, ESRF, Grenoble, France).^29^ Unexpectedly, crystals from the aerobically isolated oxidized form (prepared in the absence of formate) pressurized with 48 bar of CO_2_ in aerobic conditions resulted in a structure (HP_CO_2_) nearly identical to the Reox_120 min structure (Fig. 2) (Table S1†). The HP_CO_2_ structure shows the same SeCys dissociated form as well as clear electron density for a dioxygen/peroxide molecule coordinated to the W ion. These data show unequivocally that exposure to oxygen in the presence of either substrate results in the W–O=O⋯SeCys dissociated form, and that, contrary to previous reports,^20,21,26^ tungsten reduction is not a pre-requisite for enzyme inactivation. Remarkably, a CO_2_ molecule is present near the active site, which has never been observed before. The CO_2_ molecule is found close to the W centre (4.3 Å away) and the catalytic SeCys (4.0 Å away), being stabilized by van der Waals contacts with the carbonyl of L440 and with the dioxygen ligand, plus a hydrogen bonding interaction to the NH of G442 (Fig. 2d). Although the inactive form of the enzyme was captured, the location of the CO_2_ molecule may represent a transient affinity position along the CO_2_ diffusion pathway from the solvent to the active site, as suggested by Laun *et al.*, 2022,^30^ for the Fdh from *R. capsulatus*. This is further supported by the increased *B*-factor of the CO_2_ molecule (∼50 Å^2^), when compared with the *B*-factors of the neighbouring backbone (∼25 Å^2^) and sidechains (∼30 Å^2^).

An identical HP experiment was conducted using anaerobic crystals and revealed no discernible displacement of SeCys or any other alterations in the W site (Fig. S9†). This observation indicates that the displacement of the SeCys is not attributable solely to the applied CO_2_ pressure.

**Fig. 3:**
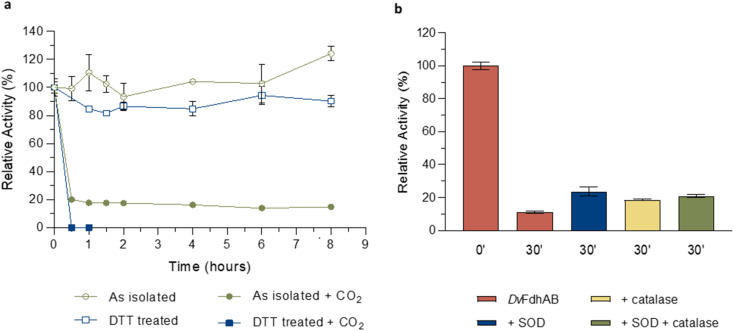
Effect of oxygen on *Dv*FdhAB formate oxidation activity in the presence of CO_2_. (a) Relative activity in the presence of oxygen with the as isolated enzyme (green) and the DTT pre-activated enzyme (blue) in the presence or absence of CO_2_ (100% activity was considered for *t* = 0 h; data without CO_2_ are from ref. 24 and 25). (b) Relative activity in the presence of formate and the ROS scavengers SOD (blue), catalase (yellow) and both (green) *versus Dv*FdhAB alone (red). Data are presented as mean values ± s.d. (*n* = 3 assay technical replicates).

**Fig. 4:**
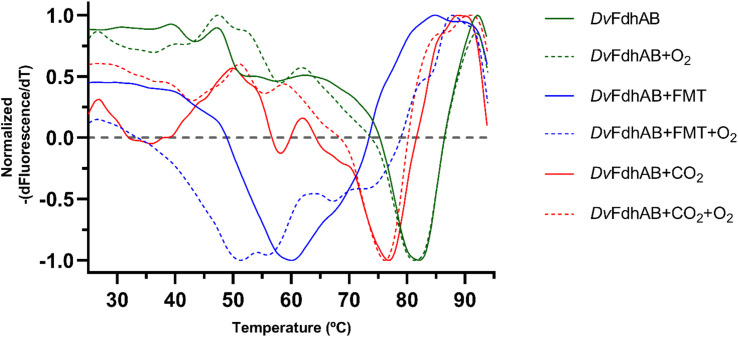
Effect of exposure and co-exposure of *Dv*FdhAB WT to formate, CO_2_ and O_2_ by thermal shift assay. The symmetric of the normalized derivative of fluorescence with respect to temperature is plotted as a function of the temperature for the assays: *Dv*FdhAB (green full, Tm = 81.4 ± 0.4 °C), *Dv*FdhAB + O_2_ (green trace, Tm = 81.5 ± 0.1 °C), *Dv*FdhAB + formate 10 mM (blue full, Tm = 60.9 ± 0.6 °C), *Dv*FdhAB + formate 10 mM + O_2_ (blue trace, Tm = 53.5 ± 1.7 °C), *Dv*FdhAB + CO_2_ (red full, Tm = 76.7 ± 0.3 °C) and *Dv*FdhAB + CO_2_+ O_2_ (red trace, Tm = 76.1 ± 0.5 °C).

The structural data from Reox_ND_NoFormate, Reox_ND_Formate, and the CO_2_ high pressure soaking (HP_CO_2_) clearly demonstrate the simultaneous requirement of oxygen and a substrate for the dissociation of SeCys from the W coordination. When *Dv*FdhAB is oxidized by O_2_ in the absence of formate (Reox_ND_NoFormate), it simply reverts to the as-isolated form and remains in that state, while when formate or CO_2_ are present along with oxygen (Reox_ND_Formate and HP_CO_2_ structures), the dissociated SeCys form emerges, suggesting that the presence of substrate induces a putative intermediate state which progresses to this form. Since the SeCys dissociated form was also obtained with exposure to oxygen in the presence of CO_2_ (HP_CO_2_), the activity of *Dv*FdhAB was also monitored in these conditions. In fact, co-exposure of a solution of non-activated *Dv*FdhAB to oxygen and CO_2_ led to 80% loss of activity in 30 min (Fig. 3a), whereas full inactivation of the DTT-activated enzyme occurs in 30 minutes, or less. This behaviour is very similar to that observed in the presence of formate and O_2_ as previously described,^26^ where DTT activated *Dv*FdhAB was almost completely inactivated after 30 minutes and completely inactivated after 1 h. A control assay, in the presence of CO_2_ in anaerobic conditions, led to a much smaller drop in the formate oxidation activity (20–30%), whereas the CO_2_ reduction activity is not affected in the same conditions (Fig. S10†). A further control of the presence of CO_2_ and O_2_ showed a decrease of about 60% in the CO_2_ reduction activity (Fig. S11†), confirming the effect of O_2_ in both reaction directions. In face of the pronounced structural changes induced by O_2_ and formate/CO_2_, Thermal Shift Assays (TSA) were performed with *Dv*FdhAB (Fig. 4). These showed that, both in the presence or absence of O_2_, the Tm of FdhAB is the same (81.5 ± 0.1 °C and 81.4 ± 0.4 °C, respectively). When formate is added in anaerobic conditions, the Tm decreases drastically, to 60.9 ± 0.6 °C and when formate and O_2_ are simultaneously present the Tm decreases even further to 53.5 ± 1.7 °C (and the thermal stability profile becomes much less sharp) (Fig. 4). These findings suggest that substrate presence induces structural changes in the enzyme, generating a more unstable species. These changes appear to facilitate O_2_-induced damage, leading to further thermal destabilisation. Regarding the presence of CO_2_ (generated by 1 M of NaHCO_3_^−^), the effect is less pronounced when compared with formate, which may be attributable to the loss of CO_2_ to the air gap above the solution, since the use of an oil layer (as used for the activity assays) would interfere with the fluorescence reading. In anaerobiosis, the Tm decrease is more moderate (76.7 ± 0.3 °C) but still significant. However, the co-exposure of FdhAB to CO_2_ and O_2_ results are not conclusive with a Tm to 76.1 ± 0.5 °C, which may be attributable to the less precise amount of CO_2_ in solution. Despite this less pronounced effect, the general trend is consistent for both formate and CO_2_. This supports the hypothesis that either substrate induces conformational changes in FdhAB, generating a more unstable, and likely more O_2_ sensitive species which is subsequently affected by O_2_.

Overall, these results confirm that, remarkably, O_2_ inactivation is promoted by the presence of either substrate and not strictly dependent on reduction of the metal, as had been previously assumed.^20,21,26^

### *Dv*FdhAB can use O_2_ as electron acceptor

To test if *Dv*FdhAB can oxidize formate using O_2_ as electron acceptor, as reported for the related *Dv*Fdh2,^23^ 1H NMR studies were used to analyse formate consumption under aerobic conditions. Using different formate concentrations (1, 10 and 50 mM), formate consumption was indeed observed for some minutes, after which the reaction stops (Fig. S12†). In the control experiment under anaerobiosis only a very small amount of formate was consumed (≈5% against ≈25% in the presence of O_2_, for 10 mM of formate) (Fig. S12†). These experiments clearly demonstrate that even in the non-activated form, *Dv*FdhAB can catalyse formate oxidation in the presence of O_2_ for a few minutes, using O_2_ as an electron acceptor. However, for longer O_2_ exposure times, *Dv*FdhAB gets inactivated, in agreement with our reported studies.^24^ The sample recovered from the NMR experiments was concentrated and the protein solution could be crystallized. Although the resulting crystals diffracted at low resolution (2.83 Å), data quality was still good enough to reveal electron density maps at the W active site in agreement with the SeCys displaced form (Fig. S13†), thus confirming the presence of this species in the inactivated enzyme. Finally, the formate oxidation activity of *Dv*FdhAB was measured with redox mediators of high redox potential, in the presence and absence of O_2_. The results (Fig. S14†) show clearly that *Dv*FdhAB has higher activity in the absence of O_2_, in contrast to *Dv*Fdh2,^23^ which is consistent with its role in anaerobic metabolism.^31^ This effect is more discernible with the *Dv*FdhAB C872A variant, which corresponds to the active form of the enzyme, equivalent to the form obtained when pre-treating the enzyme with DTT. The activity in aerobic conditions also declines gradually with time, which affects the reproducibility of the data.

Based on these data, one may propose two reoxidation pathways for D*v*FdhAB by O_2_: a “fast oxidation” likely due to the electron leakage at the FeS clusters (without damage, Reox_12 min), and a “slow oxidation” at the W site, when in the presence of substrate, potentially causing damage to the centre (Reox_120 min).

### Reactive oxygen species (ROS) are not responsible for the substrate dependent oxidative inactivation

The formation of superoxide by the FdsDABG from Cupriavidus necator was reported to be the cause of enzyme inactivation leading to the oxidation of the Mo=S sulfur and the release of sulfite.^20^ In the current work (for all W–O=O⋯SeCys structures) the sulfido ligand, when refined with full occupancy, has larger *B*-factors than the surrounding atoms (Table S3†), which may also suggest its partial replacement/loss. To investigate if ROS may play a role in the appearance of the SeCys dissociated species, crystals were incubated with different concentrations of hydrogen peroxide (H_2_O_2_). At high hydrogen peroxide concentrations (∼10 mM), for different soaking times (1–15 min), the crystals lost diffraction power (no data collection was possible). However, a 10 minute soaking with 1 mM of H_2_O_2_ yielded a 2 Å diffraction dataset and a crystal structure identical to the as-isolated (PDB_ID: 6SDR) structure, indicating that the SeCys dissociation was not promoted under these experimental conditions. Furthermore, ROS protection experiments were performed, by incubating a solution of *Dv*FdhAB with oxygen and formate in the presence and absence of two ROS scavengers (catalase and superoxide dismutase (SOD)). After 30 min of oxygen and formate exposure *Dv*FdhAB activity dropped 90% in the absence of ROS scavengers and 80% in their presence (Fig. 3b). This small difference suggests that ROS are not essential to the oxygen inactivation of *Dv*FdhAB but may contribute to it.

### Stabilization of the W(v) state was not possible after SeCys192 displacement

**Fig. 5:**
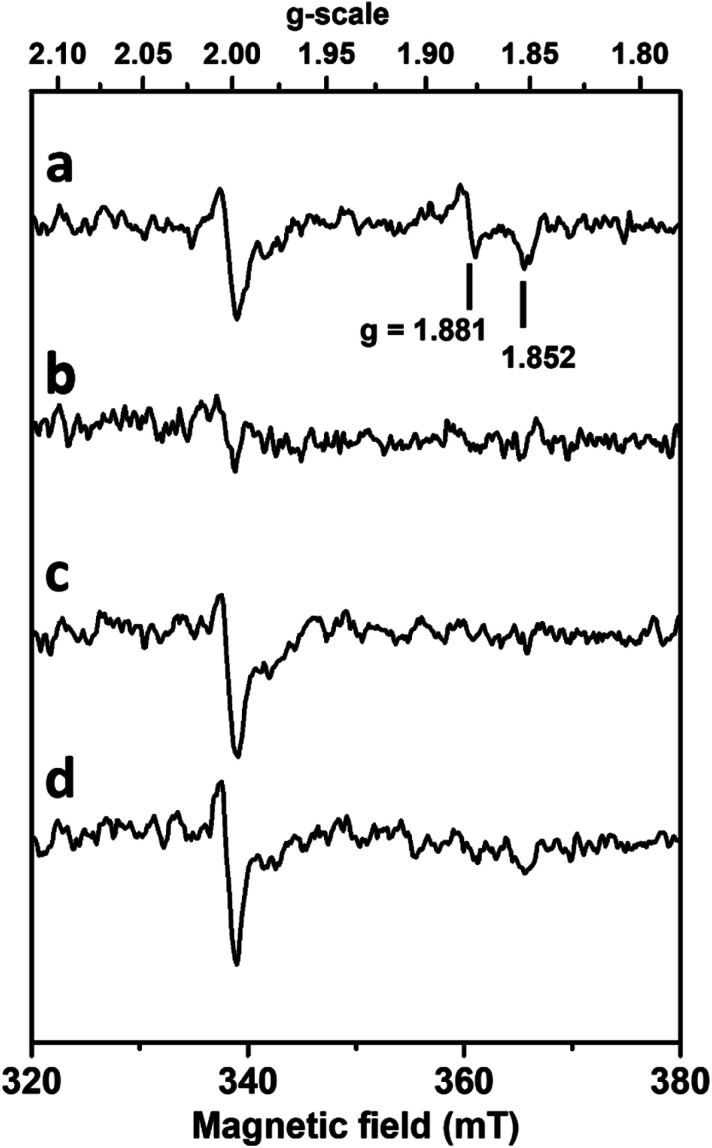
Influence of formate and oxygen exposure on W(v) EPR signals of *Dv*FdhAB. Anaerobic reduction with formate (a) followed by oxygen treatment (b), then degassing and anaerobic reduction with formate (c), and subsequent reduction with dithionite (d). EPR conditions: Temperature, 80 K; microwave power 40 mW at 9.479 GHz, modulation amplitude 1 mT at 100 kHz.

In order to analyse the influence of the structural changes identified by X-ray crystallography on the metal cofactor properties, oxygen and formate exposure assays were also monitored by EPR. Upon anaerobic reduction of the non-activated WT enzyme by formate, the intense EPR signal due to reduced [4Fe–4S]^1+^ centres develops (Fig. S15†) and the W(v)F signal at *g* = 1.995, 1.88, 1.85 is detected (Fig. 5a).

As previously discussed,^26,32^ this W(v) signal represents only 1–2% of the metal in the non-activated WT enzyme. Upon exposure to aerobic conditions, all these signals vanished (Fig. 5b) in agreement with enzyme oxidation. At lower temperature (Fig. S15†) a weak isotropic signal typical of oxidized [3Fe–4S]^1+^ clusters and representing only 2–3% in spin intensity is observed, as usually detected in as-isolated *Dv*FdhAB preparations. Upon degassing to remove O_2_ and further anaerobic reduction with formate, the spectrum of reduced FeS centres is partly recovered. This partial recovery is likely due to the decrease of *Dv*FdhAB activity caused by the co-exposure to formate and O_2_, hampering the transfer of electrons from formate to the FeS clusters. Moreover, subsequent reduction with dithionite of this formate-treated sample or of the O_2_-treated enzyme enabled to reach a reduction level of [4Fe–4S] clusters similar to that after the initial formate reduction (Fig. S15†), as reduction of FeS centres by dithionite is not dependent on enzyme activity, hence indicating that the FeS centres are not significantly affected by oxygen. In contrast, no W(v) signal is recovered after re-reduction with formate or dithionite (Fig. 5c and d).

Similar experiments performed with much longer incubation times with formate and oxygen gave essentially the same results. Thus, no new W(v) signals could be detected in the *g*-values region computed theoretically for models built from structures corresponding to inactivated enzyme (see supplementary discussion and Fig. S5 and S6, Table S5†). This indicates that the coordination sphere changes experienced by the W atom, notably the SeCys displacement and *η*^2^(O–O) ligand binding, modify the redox properties of the W cofactor and prevent the thermodynamic stabilization of the W(v) state. The stabilization of this W(v) state seems to be a specificity of the active enzyme, and substrate-dependent oxidative damage leads to an irreversibly inhibited diamagnetic W(vi) species which differs from the O_2_ insensitive W(vi) resisting state and cannot be reactivated by formate or dithionite reduction.

### Hypothesis for the inactivation mechanism

While the W–O=O⋯SeCys species reported here was never described before, a SeCys dissociated form of an Fdh was previously observed in the reanalysis of the *E. coli* Fdh-H formate-reduced structure by Raaijmakers & Romao.^13^ However, the low resolution of the data and partial disorder did not enable a proper analysis of the Mo coordination sphere, namely concerning the presence of oxygen or sulfur ligands. In addition, studies on the related *Rhodobacter capsulatus* Mo/Cys-containing Fdh,^33,34^ suggested that the Cys ligand is displaced during catalysis being replaced by an oxygen atom coordinating the Mo. This proposal was based on X-ray absorption spectroscopy studies at the Mo K-edge and NMR experiments with labelled substrates. Interestingly, these data were obtained for a formate-reduced anaerobic sample.

The data now reported raise several hypotheses regarding the SeCys displacement. Formate oxidation in the presence of O_2_ may lead to the production of ROS, which may attack the metal site leading to the SeCys displacement. This is likely to occur in the initial minutes when formate is being oxidized with O_2_ as electron acceptor but cannot explain inactivation in the presence of CO_2_, where no reductant is present. Furthermore, the experiments with ROS scavengers suggest that ROS are not essential to enzyme inactivation, although they may also contribute. A second hypothesis is that the presence of formate or CO_2_ leads to an intermediate conformational state that is more susceptible to oxidative attack, resulting in SeCys dissociation. A third hypothesis is that the dissociated form is a putative catalytic intermediate, which is trapped in the presence of oxygen. However, if the dissociated form was a catalytic intermediate, one would expect the coordination vacancy to be filled with substrate, while the SeCys192 would reoccupy its coordination position upon product release. However, there is not enough evidence to corroborate the third hypothesis, which is also challenged by the multiple time resolved and ligand soakings/co-crystallization studies previously reported^25^ that did not show SeCys dissociation in the absence of O_2_. Therefore, the most likely possibility is that the W–O=O⋯SeCys dissociated form results from a substrate-induced state where the SeCys-W coordination is more prone to attack by O_2_. The combination of the first and second hypotheses seems more coherent and can explain why this state was only captured in the experiments with oxygen and substrate, while accounting for the role of ROS. The W–O=O⋯SeCys form seems to correspond to an irreversibly inactivated state since after its formation, the enzyme could not be reactivated by removal of O_2_, DTT treatment or electrochemically by application of a low redox potential.^26^

The oxygen sensitivity of *Dv*FdhAB in the presence of substrate is in line with its protection mechanism involving an allosteric redox switch, which prevents formate binding and catalysis during transient O_2_ exposure, with *in vivo* formate concentrations in the low μM range.^26^ The current study was performed using a formate concentration of 10 mM, which is 5 times higher than the K_M_ of *Dv*FdhAB in the “protected form” (≈2 mM).^26^ Thus, under these conditions formate reduction and O_2_-dependent inactivation is expected to occur.

## Conclusions

In conclusion, the current study shows that the SeCys ligand dissociates from the W centre when *Dv*FdhAB is exposed simultaneously to oxygen and one of the substrates (formate or CO_2_), while the vacant position is occupied by a peroxide or dioxygen molecule. This form likely corresponds to a substrate-dependent oxygen/ROS inactivated form, and thus reveals a mechanism for O_2_-induced inactivation of metal-dependent Fdhs. This inactivation may also involve loss/replacement of the sulfido ligand as previously reported,^20^ including in *Dv*FdhAB.^26^ These results are also in line with previous reports on the *R*. *capsulatus* Mo-CysFdh with the presence of oxygen ligands bound to Mo, whereby Mo–O(=O) forms accompany Cys ligand dissociation.^33,34^

Regarding the dispute about the possible dissociation of the (Se)Cys ligand during catalysis, it was now demonstrated beyond doubt that this displacement does occur in the presence of oxygen and substrate. The evidence indicates that this form corresponds to an inactivated species although, with the current results, one cannot fully exclude the role of (Se)Cys dissociation in the catalytic mechanism.

A deeper understanding of the possible relation to the catalytic mechanism of Mo/W Fdh enzymes will be feasible using detailed QM-MM studies and time-resolved crystallography. Our findings are an important contribution for future efforts to engineer an enzyme envisaging the prevention of the substrate/oxygen inactivation, towards continuous catalysis under atmospheric conditions.

## Experimental

### Expression and purification of *D. vulgaris* FdhAB

*Dv*FdhAB was expressed and affinity-purified from *D. vulgaris* Hildenborough, as previously described,^24^ with some modifications. Affinity chromatography was performed with Strep-Tactin™XT 4Flow™ resin (IBA Lifesciences), and the protein eluted with 100 mM Tris/HCl buffer, pH 8, containing 150 mM NaCl and 50 mM biotin. Protein concentration was routinely determined based on *ε*_410 nm_ = 43.45 mM^−1^ cm^−1^ and purity of samples was judged by 12% SDS-polyacrylamide gel.

### Crystallization, data collection, structure solution, and refinement

Crystallization of purified wild type (WT) *Dv*FdhAB was performed in an anaerobic chamber under an argon atmosphere at <0.1 ppm of oxygen, and all the solutions were previously degassed and stored in the anaerobic chamber. All crystals were obtained using the hanging-drop vapor diffusion method, from drops of 2 μL (1 : 1, protein : precipitant ratio) in 24 well plates (24 well XRL plate Molecular Dimensions) at 20 °C. *Dv*FdhAB WT at 10 mg mL^−1^ was crystallized in conditions with 22 to 26% PEG 3350 (w/v), 0.1 M Tris–HCl pH 8.0 and 1 M LiCl, and cocrystallized with 10 mM of sodium formate^24^ with the addition of 0.2 μL of a dilution 1 : 500 from a stock of microseeds of WT *Dv*FdhAB to the drop (crystals appeared within 24 h and grew during one week).

The crystallization plates were removed from the anaerobic chamber, the wells were opened and atmospheric O_2_ was let to diffuse into the drops for different amounts of time (0 min, 12 min and 2 h) (Tables 1 and S1†).

Additionally, two control experiments were performed. Prior to oxygen exposure, crystals were transferred to two aerobic drops of harvesting solution (28% PEG 3350 (w/v), 0.1 M Tris–HCl pH 8.0 and 1 M LiCl), one with 10 mM of sodium formate and the other without sodium formate. These crystals were then further exposed to atmospheric oxygen for 34 min and 1 h, respectively. After oxygen exposure all crystals were transferred to a cryoprotectant solution consisting of the harvesting solution supplemented with 20% (v/v) glycerol, and then flash cooled in liquid nitrogen.

For the High-Pressure (HP) CO_2_ experiments, crystals were obtained aerobically using the hanging-drop vapor diffusion method, from drops of 2 μL (1 : 1, protein : precipitant ratio) in 24 well plates (24 well XRL plate Molecular Dimensions) at 20 °C. *Dv*FdhAB WT at 10 mg mL^−1^ was crystallized in conditions with 22 to 26% PEG 3350 (w/v), 0.1 M Tris–HCl pH 8.0 and 1 M LiCl^24^ with the addition of 0.2 μL of a dilution 1 : 500 from a stock of microseeds of *Dv*FdhAB WT to the drop (crystals appeared within 24 h and grew in three days). CO_2_ derivatives were prepared at the ESRF HPMX laboratory.^29,35^ The crystals were pressurized and flash-cooled in a pressure cell in two stages. First, the crystals were soaked in a pressurized atmosphere at 48 bar of CO_2_ pressure from the derivative, at room temperature, then transferred into a helium atmosphere at 48 bar at 77 K to flash-cool and stabilize the derivative. The crystals were thereafter recovered and handled in liquid nitrogen to preserve the CO_2_ derivative state.

X-ray diffraction experiments were performed at the ESRF synchrotron (on beamlines ID23-1, ID30A-3 and ID30B)^36–38^ and the data were processed with either the programs XDS^39^ and Aimless^40^ or autoPROC^41^ and Staraniso.^42^ The Staraniso software was used when data presented anisotropy, to improve overall quality of the final electron density maps. The structures were solved by molecular replacement with Phaser^43^ from the CCP4 suite,^44^ using as search model the previously published formate reduced structure (PDB ID: 6SDV), except for the CO_2_ derivative (HP_CO_2_ structure), for which the as-isolated structure (PDB ID: 6SDR) was used. The models were refined with iterative cycles of manual model building with Coot^45^ and refinement with REFMAC5.^46^ The models were rebuilt with PDBredo,^47^ the omit difference map was generated with the Phenix software^48^ and the images were produced with PyMOL.^49^ Data processing and refinement statistics are presented in Table S4.†

### Activity assays

Activity assays for formate oxidation and CO_2_ reduction were measured as previously described.^24^ Assays of oxygen exposure in the presence of CO_2_ were performed with WT *Dv*FdhAB and with DTT-activated *Dv*FdhAB. DTT activation was performed under anaerobic conditions by incubating the enzyme with 50 mM DTT for 2.5 min, followed by washing the enzyme with sample buffer (20 mM Tris–HCl, 10% glycerol and 10 mM sodium nitrate, pH 7.6) using an Amicon® Ultra Centrifugal Filter 30 MWCO. Next, both enzyme samples were diluted to 1 μM in aerobic sample buffer with 1 M sodium bicarbonate (final pH of 7.9) to a final volume of 300 μL. These samples were placed in closed flasks with mineral oil occupying the volume of the headspace (150 μL). The same procedure was followed for the control, using anaerobic buffer with bicarbonate. Additional controls were performed with WT *Dv*FdhAB where the activity was measured for CO_2_ reduction, using anaerobic and aerobic buffers. For each timepoint, a sample was collected, and formate oxidation or CO_2_ reduction activities were measured as described.^24^

For the assays with superoxide dismutase (SOD, from bovine erythrocytes, Sigma) and catalase (from bovine liver, Sigma) a similar procedure was followed, with small changes. WT *Dv*FdhAB was diluted in aerobic sample buffer with 20 mM sodium formate and without sodium nitrate. Four samples were prepared: (i) 1 μM *Dv*FdhAB, (ii) 1 μM *Dv*FdhAB and 100 U SOD, (iii) 1 μM *Dv*FdhAB and 4 nM catalase and (iv) 1 μM *Dv*FdhAB, 100 U SOD and 4 nM catalase. In samples (ii), (iii) and (iv), *Dv*FdhAB was the last enzyme added to the mixtures. The flasks were closed and incubated for 30 min. The control assay was performed with WT *Dv*FdhAB diluted in anaerobic sample buffer.

The kinetic assays under aerobic conditions were performed using WT *Dv*FdhAB and the C872A variant, which is equivalent to the active form of *Dv*FdhAB.^26^ The experiments were performed similarly to what is described for FdhAB anaerobic assays,^24^ except for the redox mediators used. In these experiments, 1 mM PMS and 100 μM DCPIP were used, instead of 2 mM BV. The reduction of DCPIP by PMS was followed at 600 nm (ε600(DCPIP) = 20.7 mM^−1^ cm^−1^). As a control, anaerobic activity was also determined using PMS and DCPIP.

### Thermal shift assays

The Thermal Shift Assays were performed using the StepOnePlus System from Applied Biosystems with an excitation wavelength of 470 nm and the ROX fluorescence emission filter (≈610 nm). Six different conditions were used: as-isolated, with sodium formate (10 mM) and with sodium hydrogencarbonate (1 M); each both in the presence and absence of atmospheric O_2_. *Dv*FdhAB (1 μM) was incubated for 10 minutes at room temperature and submitted to the assay in 250 mM potassium phosphate pH 7.0 with the Protein Thermal Shift Dye (1X, Applied Biosystems). Fluorescence was monitored and unfolding curves were generated using a temperature gradient from 25 to 95 °C in 46 min. All experiments were performed in triplicate, and the reported *T*_m_ values are based on the mean values determined from the minimum value of the first symmetric derivative of the experimental data.

### NMR experiments

NMR data were acquired at room temperature (∼293 K) on a 500 MHz Bruker NEO spectrometer (Bruker, Wissembourg, France) equipped with a 5 mm inverse detection triple-resonance z-gradient probe head (TXI) and processed using the software TopSpin 4.2.0 (Bruker BioSpin). All samples were prepared in 90%H_2_O + 10%D_2_O, 20 mM Tris–HCl buffer, pH 8 with 150 mM NaCl and 100 μM 4,4-dimethyl-4-silapentanesulfonic acid (DSS), used as a chemical shift standard. Three samples were prepared where the concentration of protein was kept at 0.5 μM and the concentration of ligand (formate) was 1, 10 and 50 mM. A fourth sample with 10 mM formate, was also prepared in anaerobic conditions. For each sample, a reference was also prepared in the same conditions but lacking the protein. All 1H spectra were acquired in a spectral window of 8196.72 Hz centred at 2354.10 Hz with 8 transients, 32 K data points, and a relaxation delay of 1.0 s. The solvent suppression was performed using an excitation sculpting scheme with gradients^50^ in which the solvent signal was irradiated with a selective pulse (Squa100.1000) with a length of 2 ms. The time for each experiment was 36 s. For each sample, 65 1H spectra were acquired, corresponding to a total of ∼40 min of reaction time.

### EPR experiments

EPR analysis was performed on a Bruker ELEXSYS E500 spectrometer equipped with an ER4102ST standard rectangular Bruker EPR cavity fitted to an Oxford Instruments ESR 900 helium flow cryostat. A sample of 0.5 mL of 100 μM WT *Dv*FdhAB enzyme in MOPS buffer was degassed in the glove box and incubated with 10 mM formate for 10 min. A first EPR sample was taken and frozen anaerobically in liquid nitrogen. Then, the enzyme solution was left under air for more than 2 hours for oxidation and a second EPR sample was taken and frozen. The enzyme solutions were then degassed again in the glove box and incubated with formate for reduction. A third EPR sample was taken and frozen anaerobically. After EPR analysis, this third sample was anaerobically thawed in the glove box and additionally reduced with 10 mM dithionite. A second set of similar experiments was performed to compare with much longer incubation time with formate (70 h) and oxygen treatment (20 h under air). All samples were studied by EPR at 15 K and 80 K to analyse the behaviour of the FeS centres and the W cofactor, respectively. Spin intensity measurements were performed by double integration of EPR spectra recorded in non-saturating conditions and comparison with a 1 mM Cu(ii)EDTA standard.

### Computational calculations of magnetic parameters

The structural models of the tungsten cofactor were created using the atomic coordinates from the Reox_120 min structure (PDB ID: 8RC9). The geometry of the models was optimized under two constraints: (i) the SSSS dihedral angle of the two dithiolates has been fixed to −33°, which correspond to the value in Reox_120 min; (ii) the positions of the tungsten and the alpha carbon of the selenocysteine have been frozen. All calculations were performed at a DFT level of theory using the B3LYP functional with the D3BJ dispersion correction.^51^ The def2-SVP basis set was chosen with the RI approximation for the geometry optimization. The determination of EPR parameters was carried out using the segmented all-electron relativistically contracted (SARC) basis set of triple-ζ quality (TZVP) for tungsten and the def2-TZVPP basis set for the other atoms.^52^ The relativistic effect was described by the ZORA method^53^ and spin–orbit couplings were considered with the SOMF treatment. All DFT calculations were performed using Orca 5.0.3 program package.^54^

## Data availability

The *Dv*FdhAB structures obtained in this work are deposited in the Protein Data Bank, under accession codes: 8RC8, 8RC9, 8RCA, 8RCB and 8RCC. Tables and figures explaining more extensively the results described in this article and additional figures presenting structural superpositions (PDF).

## Author contributions

G. V.-A., C. M. and M. J. R. conceived, designed, and analysed the resulting structures from oxygen exposure experiments in crystallo. P. C., G. V.-A., and C. M. performed and analysed the resulting structures from high pressure CO_2_ experiments in crystallo. C. M. and G. V.-A. crystallized the proteins, solved and refined all structures. G. V.-A. prepared all figures with crystal structures. R. R. M. performed protein purification, biochemical characterization, enzymatic assays and figure preparation of these results. C. M. performed thermal shift assays. A. V., G. V.-A. and C. M. conceived, designed, and performed NMR experiments. A. V. analysed and prepared images of the results from the NMR experiments. B. G. performed and analysed EPR studies. F. B. performed and analysed DFT calculations. C. M., M. J. R. and I. A. C. P. supervised the project, funded by M. J. R. and I. A. C. P. G. V.-A., C. M., M. J. R. wrote the manuscript with inputs from all other coauthors. All authors have given approval to the final version of the manuscript.

## Conflicts of interest

There are no conflicts to declare.

## Supplementary Material

SC-015-D4SC02394C-s001

## References

[cit1] Huang C. H., Tan C. S. (2014). Aerosol Air Qual. Res..

[cit2] Aresta M., Dibenedetto A., Angelini A. (2013). J. CO_2_ Util..

[cit3] Calzadiaz-Ramirez L., Meyer A. S. (2022). Curr. Opin. Biotechnol..

[cit4] Appel A. M., Bercaw J. E., Bocarsly A. B., Dobbek H., Dubois D. L., Dupuis M., Ferry J. G., Fujita E., Hille R., Kenis P. J. A., Kerfeld C. A., Morris R. H., Peden C. H. F., Portis A. R., Ragsdale S. W., Rauchfuss T. B., Reek J. N. H., Seefeldt L. C., Thauer R. K., Waldrop G. L. (2013). Chem. Rev..

[cit5] Stripp S. T., Duffus B. R., Fourmond V., Léger C., Leimkühler S., Hirota S., Hu Y., Jasniewski A., Ogata H., Ribbe M. W. (2022). Chem. Rev..

[cit6] Niks D., Hille R. (2019). Protein Sci..

[cit7] Lodh J., Roy S. (2022). J. Inorg. Biochem..

[cit8] Schwarz F. M., Schuchmann K., Müller V. (2018). Biotechnol. Biofuels.

[cit9] Kanega R., Ertem M. Z., Onishi N., Szalda D. J., Fujita E., Himeda Y. (2020). Organometallics.

[cit10] Bang J., Lee S. Y. (2018). Proc. Natl. Acad. Sci. U. S. A..

[cit11] Miller M., Robinson W. E., Oliveira A. R., Heidary N., Kornienko N., Warnan J., Pereira I. A. C., Reisner E. (2019). Angew. Chem..

[cit12] Szczesny J., Ruff A., Oliveira A. R., Pita M., Pereira I. A. C., De Lacey A. L., Schuhmann W. (2020). ACS Energy Lett..

[cit13] Raaijmakers H. C. A., Romão M. J. (2006). J. Biol. Inorg. Chem..

[cit14] Schrapers P., Hartmann T., Kositzki R., Dau H., Reschke S., Schulzke C., Leimkühler S., Haumann M. (2015). Inorg. Chem..

[cit15] Niks D., Duvvuru J., Escalona M., Hille R. (2016). J. Biol. Chem..

[cit16] Mota C. S., Rivas M. G., Brondino C. D., Moura I., Moura J. J. G., González P. J., Cerqueira N. M. F. S. A. (2011). J. Biol. Inorg. Chem..

[cit17] Cerqueira N. M. F. S. A., Fernandes P. A., Gonzalez P. J., Moura J. J. G., Ramos M. J. (2013). Inorg. Chem..

[cit18] Dong G., Ryde U. (2018). J. Biol. Inorg. Chem..

[cit19] Siegbahn P. E. M. (2022). J. Phys. Chem. B.

[cit20] Hakopian S., Niks D., Hille R. (2022). J. Inorg. Biochem..

[cit21] Gale E. F. (1939). Biochem. J..

[cit22] Enoch H. G., Lester R. L. (1975). J. Biol. Chem..

[cit23] Graham J. E., Niks D., Zane G. M., Gui Q., Hom K., Hille R., Wall J. D., Raman C. S. (2022). ACS Catal..

[cit24] Oliveira A. R., Mota C., Mourato C., Domingos R. M., Santos M. F. A., Gesto D., Guigliarelli B., Santos-Silva T., Romão M. J., Cardoso Pereira I. A. (2020). ACS Catal..

[cit25] Vilela-Alves G., Manuel R. R., Oliveira A. R., Pereira I. C., Romão M. J., Mota C. (2023). Int. J. Mol. Sci..

[cit26] Oliveira A. R., Mota C., Vilela-Alves G., Manuel R. R., Pedrosa N., Fourmond V., Klymanska K., Léger C., Guigliarelli B., Romão M. J., Cardoso Pereira I. A. (2024). Nat. Chem. Biol..

[cit27] Marangon J., Correia H. D., Brondino C. D., Moura J. J. G., Romão M. J., González P. J., Santos-Silva T. (2013). PLoS One.

[cit28] Winkler M., Duan J., Rutz A., Felbek C., Scholtysek L., Lampret O., Jaenecke J., Apfel U.-P., Gilardi G., Valetti F., Fourmond V., Hofmann E., Léger C., Happe T. (2021). Nat. Commun..

[cit29] Lafumat B., Mueller-Dieckmann C., Leonard G., Colloc’H N., Prangé T., Giraud T., Dobias F., Royant A., Van Der Linden P., Carpentier P. (2016). J. Appl. Crystallogr..

[cit30] Laun K., Duffus B., Wahlefeld S. M., Katz S., Belger D., Hildebrandt P., Mroginski M. A., Leimkühler S., Zebger I. (2022). Chem.– Eur. J..

[cit31] da Silva S. M., Voordouw J., Leitão C., Martins M., Voordouw G., Pereira I. A. C. (2013). Microbiology.

[cit32] Oliveira A. R., Mota C., Klymanska K., Biaso F., Romao M. J., Guigliarelli B., Pereira I. C. (2022). ACS Chem. Biol..

[cit33] Duffus B. R., Schrapers P., Schuth N., Mebs S., Dau H., Leimkühler S., Haumann M. (2020). Inorg. Chem..

[cit34] Kumar H., Khosraneh M., Bandaru S. S. M., Schulzke C., Leimkühler S. (2023). Molecules.

[cit35] Van Der Linden P., Dobias F., Vitoux H., Kapp U., Jacobs J., Mc Sweeney S., Mueller-Dieckmann C., Carpentier P. (2014). J. Appl. Crystallogr..

[cit36] Nurizzo D., Mairs T., Guijarro M., Rey V., Meyer J., Fajardo P., Chavanne J., Biasci J. C., McSweeney S., Mitchell E. (2006). J. Synchrotron Radiat..

[cit37] McCarthy A. A., Barrett R., Beteva A., Caserotto H., Dobias F., Felisaz F., Giraud T., Guijarro M., Janocha R., Khadrouche A., Lentini M., Leonard G. A., Lopez Marrero M., Malbet-Monaco S., McSweeney S., Nurizzo D., Papp G., Rossi C., Sinoir J., Sorez C., Surr J., Svensson O., Zander U., Cipriani F., Theveneau P., Mueller-Dieckmann C. (2018). J. Synchrotron Radiat..

[cit38] Von Stetten D., Carpentier P., Flot D., Beteva A., Caserotto H., Dobias F., Guijarro M., Giraud T., Lentini M., McSweeney S., Royant A., Petitdemange S., Sinoir J., Surr J., Svensson O., Theveneau P., Leonard G. A., Mueller-Dieckmann C. (2020). J. Synchrotron Radiat..

[cit39] Kabsch W. (2009). Acta Crystallogr., Sect. D: Biol. Crystallogr..

[cit40] Evans P. R., Murshudov G. N. (2013). Acta Crystallogr., Sect. D: Biol. Crystallogr..

[cit41] Vonrhein C., Flensburg C., Keller P., Sharff A., Smart O., Paciorek W., Womack T., Bricogne G. (2011). Acta Crystallogr., Sect. D: Biol. Crystallogr..

[cit42] Vonrhein C., Tickle I. J., Flensburg C., Keller P., Paciorek W., Sharff A., Bricogne G. (2018). Acta Crystallogr., Sect. A: Found. Adv..

[cit43] Mccoy A. J., Grosse-kunstleve R. W., Adams P. D., Winn M. D., Storoni L. C., Read R. J. (2007). J. Appl. Crystallogr..

[cit44] Winn M. D., Charles C., Cowtan K. D., Dodson E. J., Leslie A. G. W., Mccoy A., Stuart J., Garib N., Powell H. R., Randy J. (2011). Acta Crystallogr., Sect. D: Biol. Crystallogr..

[cit45] Emsley P., Lohkamp B., Scott W. G., Cowtan K. (2010). Acta Crystallogr., Sect. D: Biol. Crystallogr..

[cit46] Vagin A. A., Steiner R. A., Lebedev A. A., Potterton L., McNicholas S., Long F., Murshudov G. N. (2004). Acta Crystallogr., Sect. D: Biol. Crystallogr..

[cit47] Joosten R. P., Salzemann J., Bloch V., Stockinger H., Berglund A. C., Blanchet C., Bongcam-Rudloff E., Combet C., Da Costa A. L., Deleage G., Diarena M., Fabbretti R., Fettahi G., Flegel V., Gisel A., Kasam V., Kervinen T., Korpelainen E., Mattila K., Pagni M., Reichstadt M., Breton V., Tickle I. J., Vriend G. (2009). J. Appl. Crystallogr..

[cit48] Liebschner D., Afonine P. V., Baker M. L., Bunkoczi G., Chen V. B., Croll T. I., Hintze B., Hung L. W., Jain S., McCoy A. J., Moriarty N. W., Oeffner R. D., Poon B. K., Prisant M. G., Read R. J., Richardson J. S., Richardson D. C., Sammito M. D., Sobolev O. V., Stockwell D. H., Terwilliger T. C., Urzhumtsev A. G., Videau L. L., Williams C. J., Adams P. D. (2019). Acta Crystallogr., Sect. D: Struct. Biol..

[cit49] The PyMOL Molecular Graphics System, Version 3.0, Schrödinger, LLC, 2019

[cit50] Hwang T.-L., Shaka A. J. (1995). J. Magn. Reson., A.

[cit51] Grimme S. (2004). J. Comput. Chem..

[cit52] Weigend F., Ahlrichs R. (2005). Phys. Chem. Chem. Phys..

[cit53] Van Lenthe E., Baerends E. J., Snijders J. G. (1993). J. Chem. Phys..

[cit54] Neese F. (2022). Wiley Interdiscip. Rev.: Comput. Mol. Sci..

