# Supplementary material for: Substrate-dependent oxidative inactivation of a W-dependent formate dehydrogenase involving selenocysteine displacement
Source: Chem Sci. 2024 Jul 16;15(32):13090–101. doi: 10.1039/d4sc02394c (PMC11323313; doi:10.1039/d4sc02394c)
Supplement: SC-015-D4SC02394C-s001 [file SC-015-D4SC02394C-s001.pdf]

Electronic Supplementary Material (ESI) for Chemical Science.
This journal is © The Royal Society of Chemistry 2024

Supplementary Information

Substrate-dependent oxidative inactivation of a W-dependent formate dehydrogenase involving selenocysteine displacement.

Guilherme Vilela-Alves[1,2], Rita R. Manuel[3], Aldino Viegas[1,2], Philippe Carpentier[4,5], Frederic Biaso[6], Bruno Guigliarelli[6], Inês A. C. Pereira[3]*, Maria João Romão[1,2]* and Cristiano Mota[1,2]*

[1]Associate Laboratory i4HB – Institute for Health and Bioeconomy, NOVA School of Science and Technology, Universidade NOVA de Lisboa, 2829-516 Caparica, Portugal

[2]UCIBIO, Applied Molecular Biosciences Unit, Department of Chemistry, NOVA School of Science and Technology, Universidade NOVA de Lisboa, 2829-516 Caparica, Portugal

[3]Instituto de Tecnologia Química e Biológica António Xavier, Universidade Nova de Lisboa, Av. da República, 2780-157 Oeiras, Portugal

[4]European Synchrotron Radiation Facility, Grenoble, France

[5]Institut de Recherche Interdisciplinaire de Grenoble (IRIG), Laboratoire Chimie et Biologie des Métaux (LCBM), Université Grenoble Alpes, CNRS, CEA, Grenoble, France

[6]Aix Marseille Univ, CNRS, BIP, Laboratoire de Bioénergétique et Ingénierie des Protéines, Marseille 13402, France

*Corresponding authors: mjr@fct.unl.pt, cd.mota@fct.unl.pt, ipereira@itqb.unl.pt

Index

Supplementary Discussion: Computational calculations of magnetic parameters..............................3

Table S 1- Description of the procedure used to obtain the different structures of *Dv*FdhAB in this and other works. . 4
Table S 2- Distances between the W ion, Se atom of SeCys and the (di)oxygen ligand atom(s) for the Control_Red , Reox_120min, Reox_ND_NoFormate, Reox_ND_Formate and HP_CO_2 structures (with respective crystallographic resolutions) and of the optimized W^V structural models (Figure S12). 5
Table S 3- B-factor values for the W ion, sulfido ligand (S), the four sulfur atoms of the 2 dithiolenes, Se atom of SeCys and the two atoms of the dioxygen molecule for the Control_Red, Reox_12min, Reox_120min, Reox_ND_No_Formate, Reox_ND_Formate and HP_CO_2 structures and respective resolutions.................... 6
Table S 4- Crystallographic data processing and refinement statistics. 7
Table S 5- DFT-predicted g-values for the optimized W^V structural models (Figure S12). 10

Figure S 1- Superposition of *Dv*FdhAB WT formate reduced (PDB_ ID: 6SDV) (green) and Control_Red structure (orange) in two different views. 11
Figure S 2- Superposition of *Dv*FdhAB WT WT as-isolated (oxidized) (PDB_ID: 6SDR) (red), formate reduced (green) and oxygen exposed Reox_12min structure (yellow) in two different views. 12
Figure S 3- Solved, unrefined, structure obtained from the *Dv*FdhAB WT crystals co-crystalized with formate and exposed to air for 20 min. 13
Figure S 4- Alternative modelling of different ligands for the electron density between W and Se in the *Dv*FdhAB Reox_120min structures. 14
Figure S 5- Structural models of the W^V cofactor used for DFT calculations........................ 15
Figure S 6- Optimized geometry of the W^V models obtained by DFT calculations. 16
Figure S 7- Comparison of the metal coordination between the dioxygen/peroxide containing active sites of *Dv*FdhAB Reox_120min (cyan) and *Dg*AOR H2O2-soaked (PDB_ID: 4C80) (brown). 17
Figure S 8- Superposition of *Dv*FdhAB WT as-isolated (oxidized) (PDB_ID: 6SDR) (red) and oxygen exposed Reox_ND_NoFormate structure (blue) in two different views. 18
Figure S 9- Control HP experiment with anaerobic *Dv*FdhAB crystals under CO_2 pressure. 19
Figure S 10- Relative activity for CO_2 reduction (red) and formate oxidation (blue) of as-isolated *Dv*FdhAB incubated in anaerobic conditions in the presence of CO_2. 20
Figure S 11- Relative activity for CO_2 reduction of as-isolated *Dv*FdhAB incubated in aerobic conditions in the presence of CO_2. 21
Figure S 12- *Dv*FdhAB activity assays by 1H NMR. 22
Figure S 13- Solved, unrefined, structure (yellow) obtained from the *Dv*FdhAB WT crystals produced with the concentrated solution left from the NMR experiments with formate under air, at a resolution of 2.83 Å. 23
Figure S 14- Formate oxidation activity measured in aerobic and anaerobic conditions, of as-isolated WT *Dv*FdhAB and C872A variant (this variant is equivalent to the DTT-activated form [4]). 24
Figure S 15- Influence of formate and oxygen exposure on FeS center EPR signals of *Dv*FdhAB. 25

References........................26

Supplementary Discussion: DFT calculations of the potential influence of SeCys displacement on the W(V) cofactor g-values

As the g-values of W(V) species can range in a very broad domain and to help detection of potentially new W(V) EPR signals, a quantum chemistry study was performed on several W(V) cofactor models to evaluate the potential influence of SeCys displacement and the binding of oxygenated species on the W(V) g-tensor. Eight structural models (numbered 1 to 8) (Figure S5 and S6) were built based on the structure of the formate and oxygen-treated DvFdhAB (Reox_120min). These models have in common the coordination of W(V) ion by the four sulfur atoms of the two pterins, a η^2(O-O) or water ligand and a displaced SeCys, while they differ in the nature of the exogenous ligand (sulfur or oxygen), the protonation state of the cofactor and possible H-bonds between a η^2(O-O) or water ligand and sulfur and/or selenium atoms (Figure S5 and S6). Optimized geometry consistent with protein surrounding constraints could be obtained only for structural models 1-3 and 7. The g-values of W(V) species were calculated for these models. This gives values ranging from about g = 2.1 to 2.6 (Table S5) for models 1-3, much higher than the values observed experimentally for the W(V)F and W(V)D species and calculated for models with bound SeCys ligand.[1] Moreover, the g-values calculated for model 7 are much lower than g=2.00 and does not fit with those of W(V)F and W(V)D species. Thus, in complement to models investigated in previous work,[1] these DFT calculations provide additional support to show that the experimentally detected W(V)F and W(V)D species associated to active DvFdh does not correspond to species with non-coordinated SeCys.

Table S 1- Description of the procedure used to obtain the different structures of *Dv*FdhAB in this and other works.

Structure	Procedure
As-isolated [2]<br>PDB_ID: 6SDR	*Dv*FdhAB crystallized **as-isolated** in the presence of O_2 (As-isolated).
Formate-reduced [2]<br>PDB_ID: 6SDV	*Dv*FdhAB co-crystallized with 10 mM of sodium formate and **not exposed** to O_2 (Reduced).
Control_Red<br>PDB_ID: 8RC8	*Dv*FdhAB co-crystallized with 10 mM of sodium formate and **not exposed** to O_2 (Reduced).
Reox_12min [3]<br>PDB_ID: 8BQL	*Dv*FdhAB co-crystallized with 10 mM of sodium formate; plate well opened and crystals exposed to atmospheric O_2, for **12 min**, while still in the original drop (which **contains** 10 mM of sodium formate), and then flash cooled in liquid nitrogen (as-isolated/Oxidized).
Reox_120min<br>PDB_ID: 8RC9	*Dv*FdhAB co-crystallized with 10 mM of sodium formate; plate well opened and the crystals were exposed to atmospheric O_2, for **120 min**, while still in the original drop (which **contains** 10 mM of sodium formate), and then flash cooled in liquid nitrogen (W-O=O…SeCys form).
Reox_ND_NoFormate<br>PDB_ID: 8RCA	*Dv*FdhAB co-crystallized with 10 mM of sodium formate; the crystals were transferred from the original drop to a **N**ew **D**rop (oxygenated), mimicking the mother liquor but **without** sodium formate, and were exposed to atmospheric O_2, for 60 min, and then flash cooled in liquid nitrogen (as-isolated/Oxidized).
Reox_ND_Formate<br>PDB_ID: 8RCB	*Dv*FdhAB co-crystallized with 10 mM of sodium formate; the crystals were transferred from the original drop to a **N**ew **D**rop (oxygenated), mimicking the mother liquor **containing** 10 mM of sodium formate, and were exposed to atmospheric O_2, for 34 min, and then flash cooled in liquid nitrogen (W-O=O…SeCys form).
HP_CO_2<br>PDB_ID: 8RCC	*Dv*FdhAB aerobically crystallized **without** sodium formate; the crystals were pressurised with 48 bar of CO_2, and then flash cooled in liquid nitrogen, under 200 bar helium (W-O=O…SeCys form).

Table S 2- Distances between the W ion, Se atom of SeCys and the (di)oxygen ligand atom(s) for the Control_Red , Reox_120min, Reox_ND_NoFormate, Reox_ND_Formate and HP_CO_2 structures (with respective crystallographic resolutions) and of the optimized W^V structural models (Figure S12).

Bond length and distance (in Å)	**W-S$_{sulfido}$**	**W-O$_{dioxygen}$**	**W---Se**	**O-O$_{dioxygen}$**	**O---Se**
Control_Red (2.00 Å)(8RC8)	2.31	n.a.	2.56	n.a.	n.a.
Reox_120min (2.06 Å)(8RC9)	2.17	2.34 2.52	4.20	1.23	2.46 2.57
Reox_ND_NoFormate (1.66 Å)(8RCA)	1.96	n.a.	2.60	n.a.	n.a.
Reox_ND_Formate (2.11 Å)(8RCB)	2.27	2.32 2.64	4.48	1.21	2.62 3.01
HP_CO_2 (2.30 Å)(8RCC)	2.82	2.43 1.93	3.69	1.22	2.19 2.89
Model **1**	2.204	1.968 2.496	4.792	1.406	2.673 3.525
Model **2**	2.168	2.805 2.128	5.400	1.298	3.606 4.127
Model **3**	2.470	1.946 2.038	4.556	1.428	2.882 3.791
Model **7**	2.174	2.237 (water)	4.925	n.a.	3.081

Table S 3- B-factor values for the W ion, sulfido ligand (S), the four sulfur atoms of the 2 dithiolenes, Se atom of SeCys and the two atoms of the dioxygen molecule for the Control_Red, Reox_12min, Reox_120min, Reox_ND_No_Formate, Reox_ND_Formate and HP_CO_2 structures and respective resolutions.

B-factor (Å^2)	Control_Red (2.00 Å)	Reox_12min (1.91 Å)	Reox_120min (2.06 Å)	Reox_ND_No_Formate (1.66 Å)	Reox_ND_Formate (2.11 Å)	HP_CO_2 (2.30 Å)
**W**	24.09	24.75	35.67	21.05	33.75	30.77
**S**	28.66	26.61	58.88	18.74	60.37	43.89
**S12**$_{MGD1}$	22.13	21.90	29.10	21.74	24.24	26.59
**S13**$_{MGD1}$	21.80	24.55	30.23	21.88	24.30	27.26
**S12**$_{MGD2}$	24.31	26.19	32.19	21.22	37.30	29.81
**S13**$_{MGD2}$	23.65	25.26	36.35	20.72	29.61	26.54
**Se**	25.54	29.30	53.55	25.10	92.48	75.80
**Cβ**$_{U192}$	27.58	30.10	49.66	24.37	55.09	56.78
**Cα**$_{U192}$	28.29	29.85	44.20	24.09	40.43	49.87
**O1**	NA	NA	44.42	NA	25.44	32.52
**O2**	NA	NA	32.91	NA	39.70	33.80

Table S 4- Crystallographic data processing and refinement statistics.

**Crystal**	**Control_Red**	**Reox_120min**	**Reox_120min**_Staraniso
**PDB$_{code}$**	8RC8		8RC9
**Diffraction Data**			
Space group	$P2_12_12_1$	$P2_12_12_1$	$P2_12_12_1$
Cell dimensions (Å) (º)	a=64.99, b=124.35, c=149.90	a=65.18, b=127.93, c=149.47	a=65.18, b=127.93, c=149.47
Wavelength (Å)	0.9686	0.9686	0.9686
Beamline	ESRF ID30B	ESRF ID30B	ESRF ID30B
No. Crystals	1	1	1
Resolution range of data (Å) (last shell)	47.85 – 2.00 (2.04 – 2.00)	97.19 – 2.28 (2.32 – 2.28)	97.19 – 2.06 (2.15 – 2.06)
Completeness (%) (last shell)	99.60 (92.40)	96.52 (98.31)	87.87 (49.15)
Rmerge (last shell)	0.182 (1.224)	0.110 (0.658)	0.119 (1.110)
Rmeas (last shell)	0.214 (1.429)	0.127 (0.758)	0.137 (1.241)
I/σI (last shell)	6.6 (1.3)	7.9 (2.1)	7.0 (1.5)
CC 1/2 (last shell)	0.994 (0.527)	0.991 (0.632)	0.991 (0.495)
Redundancy (last shell)	7.0 (6.6)	4.1 (4.2)	4.3 (5.1)
**Refinement**			
Reflections used in refinement (work (free))	77848 (4152)		65087 (3244)
Rwork	0.183		0.206
Rfree	0.219		0.255
Nº of non-hydrogen atoms	9804		9587
Protein	9205		9224
Ligands	175		154
Ions	2		2
Solvent	422		207
**Geometry and *B*-factors**			
RMSD bond lengths (Å)	0.004		0.005
RMSD bond angles (º)	1.052		1.393
Average B-factor ALL (Å^2)	31.24		40.72
Protein (Å^2)	33.28		43.64
Ligands (Å^2)	31.06		34.28
Ions (Å^2)	26.38		47.27
Solvent (Å^2)	33.34		33.82
Ramachandran favoured (%)	96.32		95.47
Ramachandran outliers (%)	0.17		0.34
Molprobity score	1.26		1.50
Clashscore	2.38		2.98

**Crystal**	**Reox_ND_NoFormate**	**Reox_ND_Formate**	**Reox_ND_Formate**_Staraniso
**PDB_{code}**	8RCA		8RCB
**Diffraction Data**			
Space group	$P2_12_12_1$	$P2_12_12_1$	$P2_12_12_1$
Cell dimensions (Å) (º)	a=64.56, b=128.01, c=148.91	a=64.43, b=123.09, c=148.14	a=64.43, b=123.09, c=148.14
Wavelength (Å)	0.7749	0.9686	0.9686
Beamline	ESRF ID23-1	ESRF ID30B	ESRF ID30B
No. Crystals	1	1	1
Resolution range of data (Å) (last shell)	97.26 – 1.66 (1.79 – 1.66)	94.67 – 2.42 (2.46 – 2.42)	94.67 – 2.11 (2.28 – 2.11)
Completeness (%) (last shell)	90.60 (53.30)	98.64 (98.80)	91.15 (40.48)
Rmerge (last shell)	0.104 (1.098)	0.140 (1.070)	0.153 (1.187)
Rmeas (last shell)	0.117 (1.201)	0.149 (1.132)	0.163 (1.279)
I/σI (last shell)	7.9 (1.5)	10.4 (2.1)	8.4 (1.6)
CC 1/2 (last shell)	0.996 (0.658)	0.996 (0.774)	0.996 (0.591)
Redundancy (last shell)	4.66 (6.12)	9.0 (9.4)	8.9 (7.3)
**Refinement**			
Reflections used in refinement (work (free))	102948 (5448)		54095 (2723)
Rwork	0.196		0.199
Rfree	0.232		0.240
Nº of non-hydrogen atoms	9971		9410
Protein	9228		9153
Ligands	172		161
Ions	2		2
Solvent	569		94
**Geometry and *B*-factors**			
RMSD bond lengths (Å)	0.008		0.003
RMSD bond angles (º)	1.511		1.212
Average B-factor ALL ($Å^2$)	30.65		38.68
Protein ($Å^2$)	32.60		41.18
Ligands ($Å^2$)	25.27		33.93
Ions ($Å^2$)	19.89		47.06
Solvent ($Å^2$)	33.35		28.54
Ramachandran favoured (%)	96.33		95.85
Ramachandran outliers (%)	0.26		0.17
Molprobity score	1.54		1.20
Clashscore	4.43		1.63

**Crystal**	**HP_CO_2**	**HP_CO_2**_Staraniso
**PDB$_{code}$**		8RCC
**Diffraction Data**		
Space group	$P2_12_12_1$	$P2_12_12_1$
Cell dimensions (Å) (º)	a=64.81, b=124.40, c=150.31	a=64.81, b=124.40, c=150.31
Wavelength (Å)	0.9677	0.9677
Beamline	ESRF ID30A-3	ESRF ID30A-3
No. Crystals	1	1
Resolution range of data (Å) (last shell)	95.84 – 2.66 (2.70 – 2.66)	95.84 – 2.30 (2.44 – 2.30)
Completeness (%) (last shell)	96.26 (99.05)	89.19 (45.52)
Rmerge (last shell)	0.194 (0.901)	0.239 (1.421)
Rmeas (last shell)	0.214 (0.995)	0.264 (1.546)
I/σI (last shell)	8.1 (2.1)	6.5 (1.4)
CC 1/2 (last shell)	0.986 (0.639)	0.985 (0.465)
Redundancy (last shell)	5.5 (5.5)	5.6 (6.3)
**Refinement**		
Reflections used in refinement (work (free))		42746 (2267)
Rwork		0.194
Rfree		0.244
Nº of non-hydrogen atoms		9544
Protein		9193
Ligands		246
Ions		2
Solvent		103
**Geometry and *B*-factors**		
RMSD bond lengths (Å)		0.005
RMSD bond angles (º)		1.360
Average B-factor ALL (Å^2)		33.54
Protein (Å^2)		35.93
Ligands (Å^2)		35.28
Ions (Å^2)		37.33
Solvent (Å^2)		25.44
Ramachandran favoured (%)		94.86
Ramachandran outliers (%)		0.34
Molprobity score		1.93
Clashscore		5.17

Table S 5- DFT-predicted g-values for the optimized W^V structural models (Figure S12).

Model	Calculated g-values
1	2.189 2.049 2.026
2	2.019 2.013 2.013
3	2.616 2.171 2.057
4	n.d. [1]
5	n.d. [1]
6	n.d. [1]
7	1.931 1.927 1.891
8	n.d. [1]

[1] for these models, the optimized structure cannot fit with the rest of the protein due to strong distortions of dithiolene moieties.

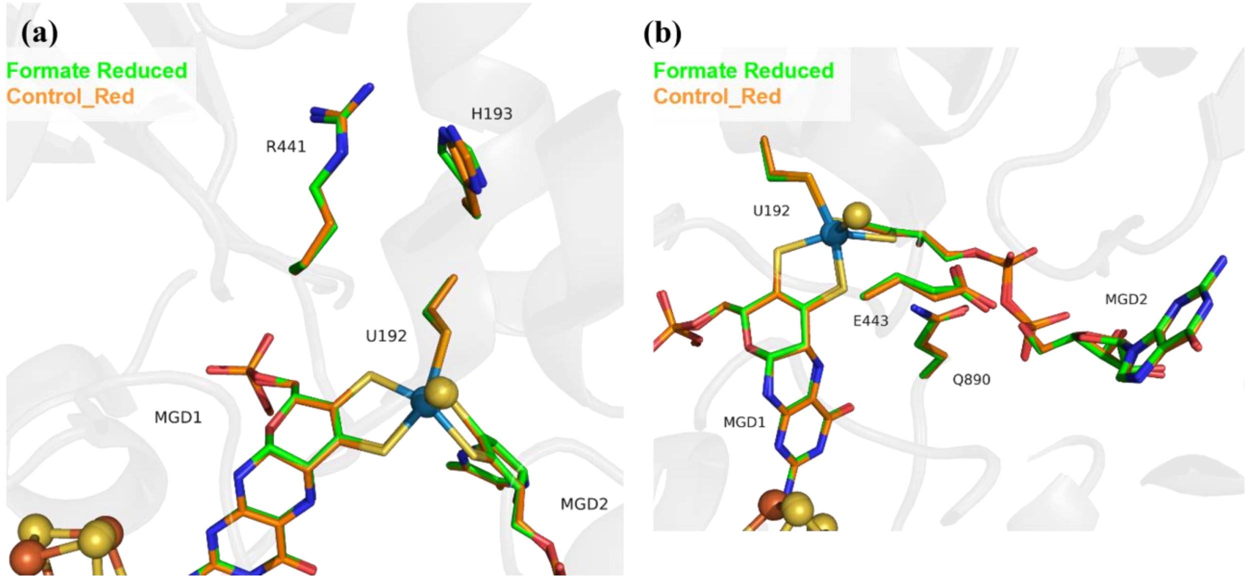

Figure S 1- Superposition of *Dv*FdhAB WT formate reduced (PDB_ ID: 6SDV) (green) and Control_Red structure (orange) in two different views.
Rmsd is 0.21 Å for 964 Cα atoms of *Dv*FdhA, and of 0.19 Å for 214 Cα atoms of *Dv*FdhB. **(a)** U192, H193, R441 and the two MGD co-factors are shown as sticks. **(b)** U192, E443, Q890 and the two MGD co-factors are shown as sticks.

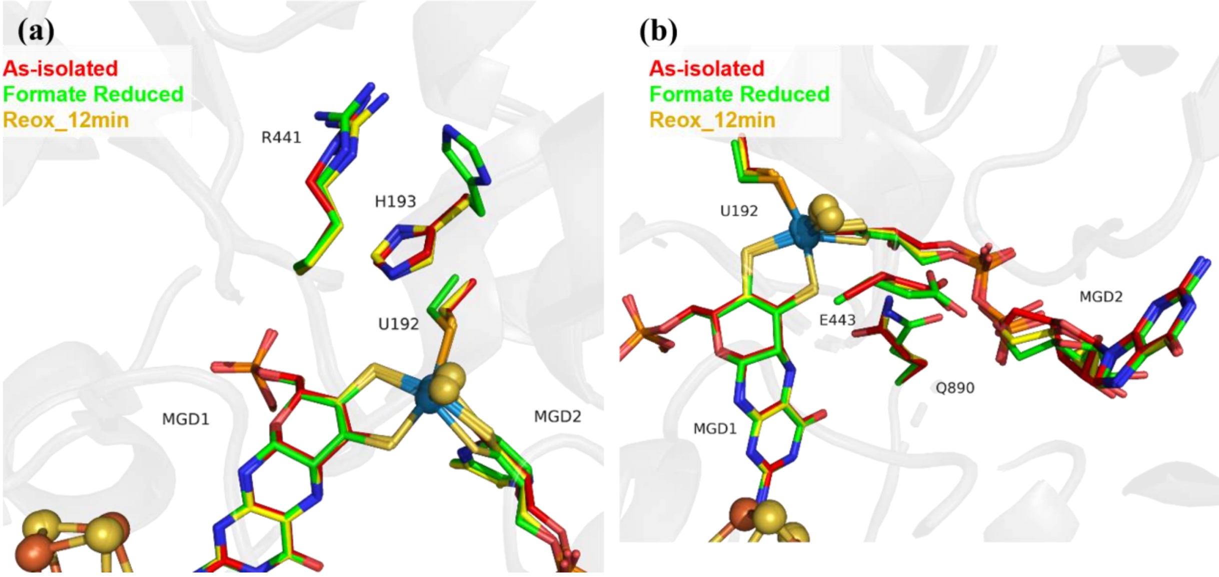

Figure S 2- Superposition of *Dv*FdhAB WT WT as-isolated (oxidized) (PDB_ID: 6SDR) (red), formate reduced (green) and oxygen exposed Reox_12min structure (yellow) in two different views. Rmsd is 0.15 Å for 963 Cα atoms of *Dv*FdhA, and of 0.17 Å for 214 Cα atoms of *Dv*FdhB. **(a)** U192, H193, R441 and the two MGD co-factors are shown as sticks. **(b)** U192, E443, Q890 and the two MGD co-factors are shown as sticks.

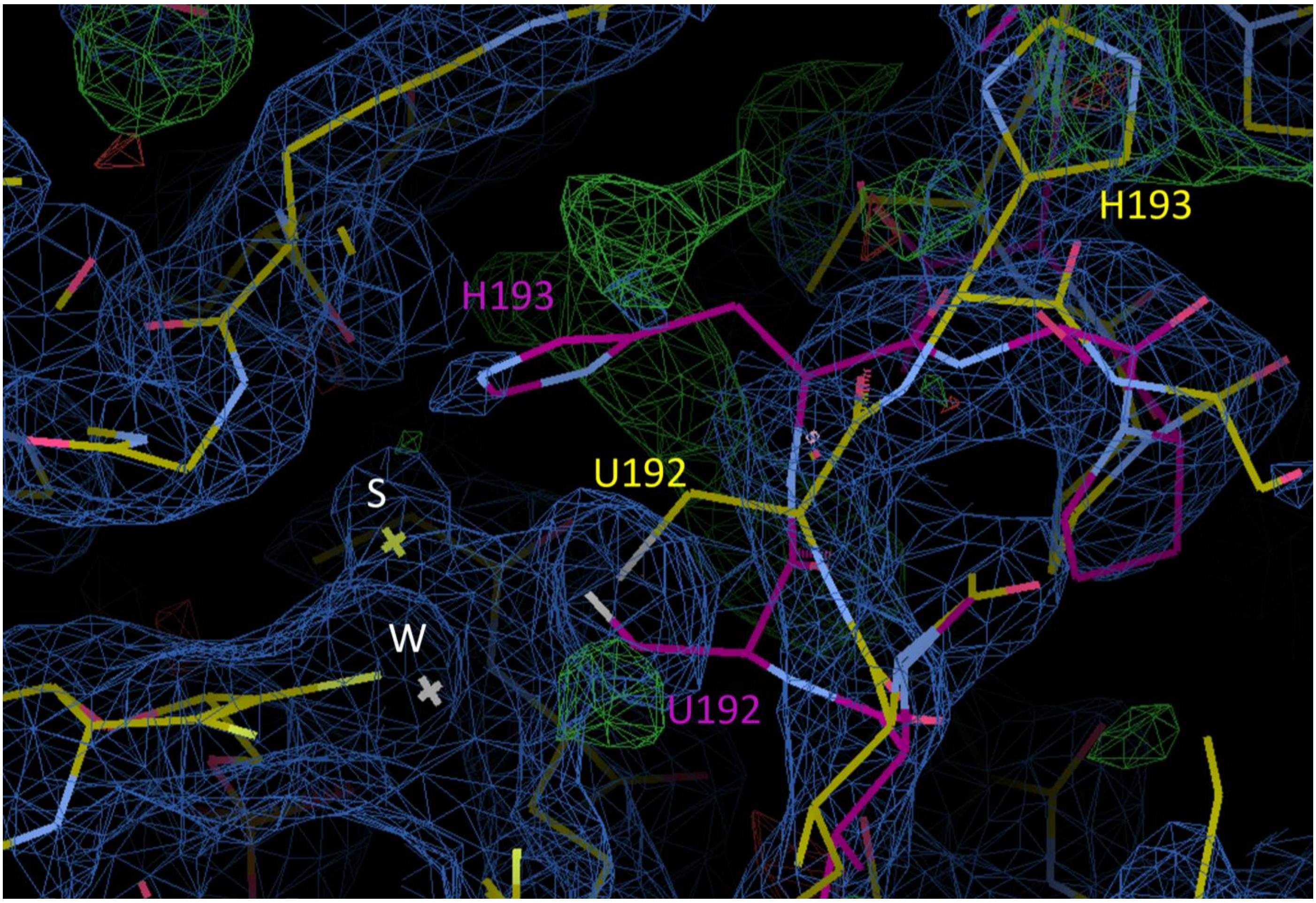

Figure S 3- Solved, unrefined, structure obtained from the *Dv*FdhAB WT crystals co-crystalized with formate and exposed to air for 20 min.
*Dv*FdhAB 20 min structure displaying a mixture of states (50%/50%) of both WT as isolated (oxidized) (magenta) (PDB_ID: 6SDR) and Reox_120min (yellow), near the active site (I191-S194). 2Fo-Fc maps at 1 σ (blue mesh) and Fo-Fc maps at 3 σ (green and red mesh, respectively for positive and negative densities) are shown. Image produced with Coot [4].

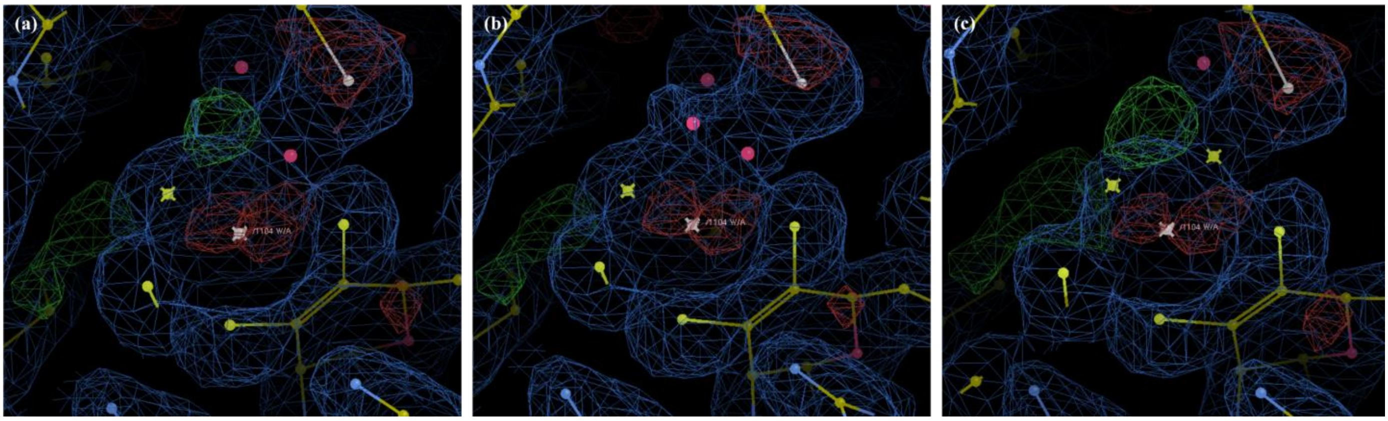

Figure S 4- Alternative modelling of different ligands for the electron density between W and Se in the *Dv*FdhAB Reox_120min structures.

The W ion (grey cross), SeCys Se atom (grey sphere), the two MGD co-factors (yellow lines), sulfido ligand (yellow cross) and water molecules (red sphere) are shown. 2Fo-Fc electron density map contoured at 1σ (blue mesh) and Fo-Fc map contoured at 3σ (green and red mesh, respectively for positive and negative density) are shown. Images produced with Coot [4]. **(a)** Modelling hypothesis with one water molecule at full occupancy. **(b)** Modelling hypothesis with two water molecules, each at half occupancy. **(c)** Modelling hypothesis with two sulfido ligands, each at half occupancy.

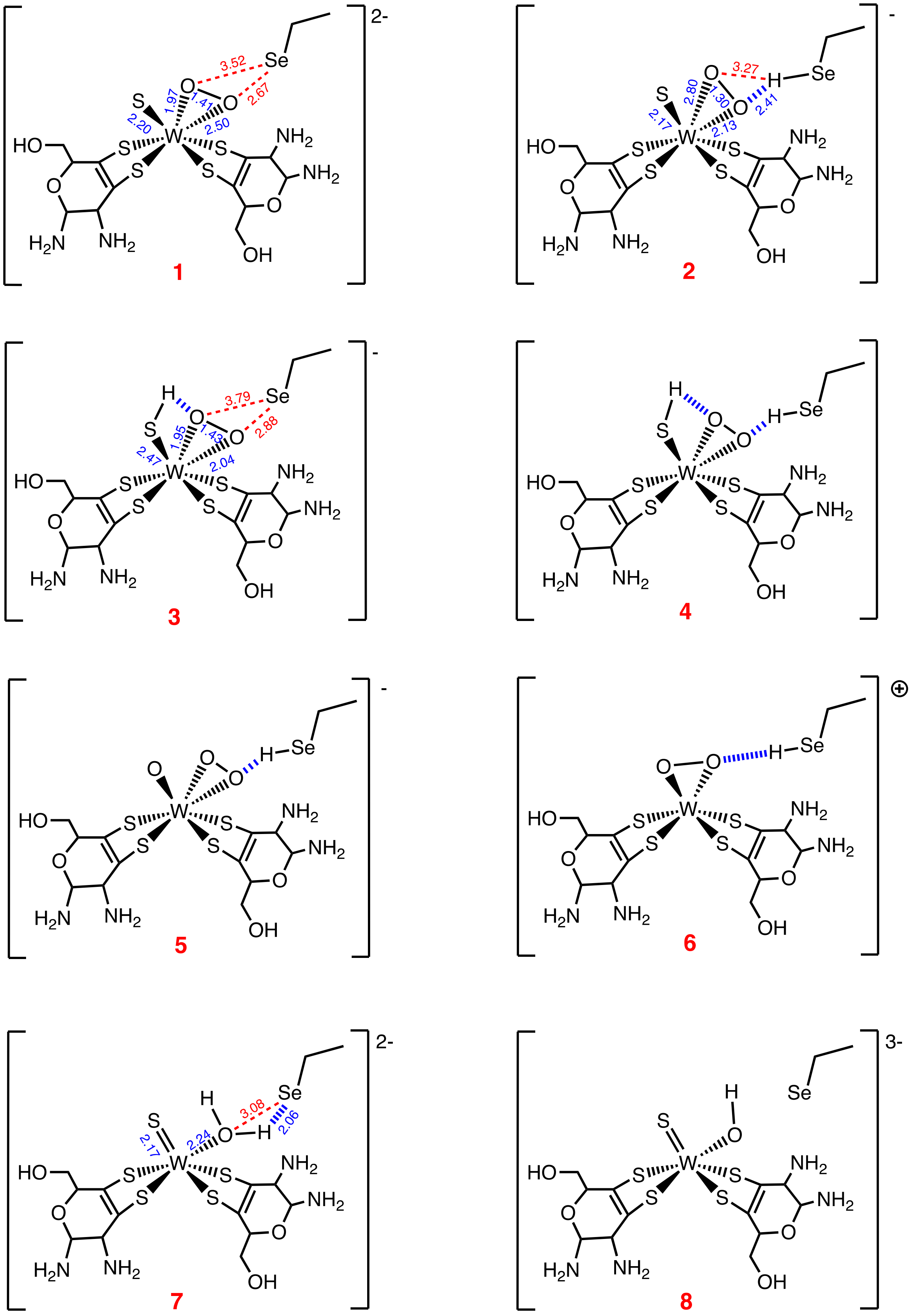

Figure S 5- Structural models of the W^{V} cofactor used for DFT calculations.
Interatomic distances were indicated, in Å, only for models leading to optimized geometry consistent with protein surrounding constraints.

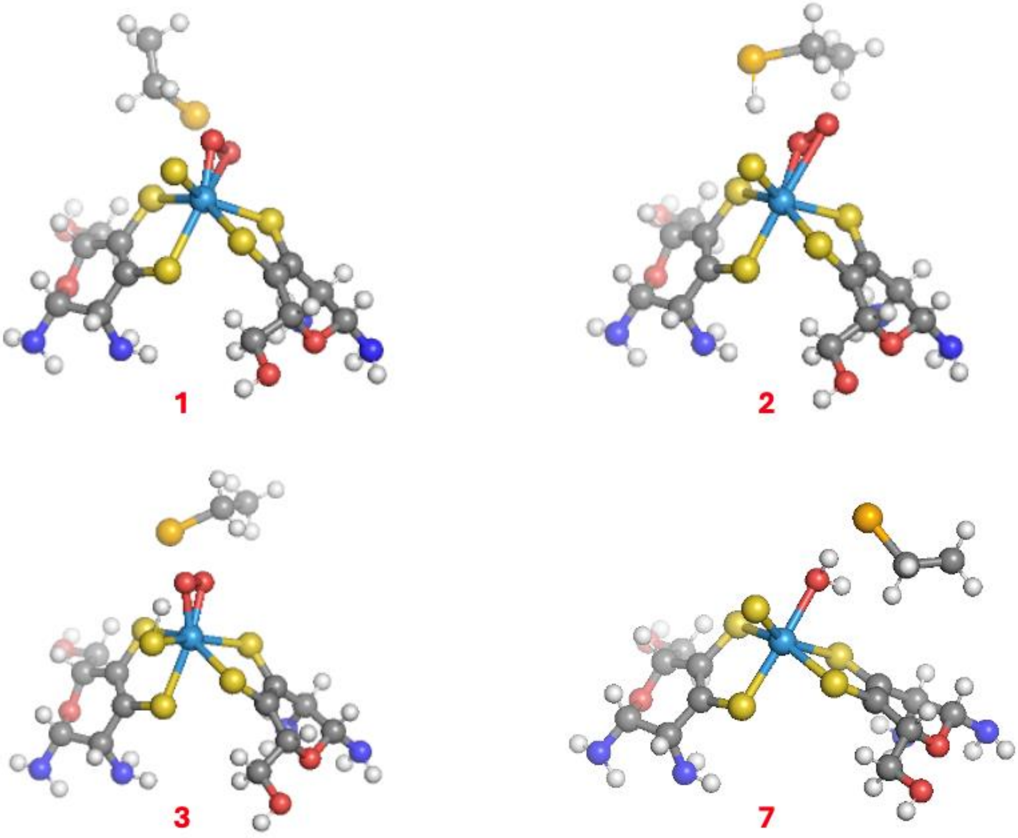

Figure S 6- Optimized geometry of the W^{V} models obtained by DFT calculations.

The W ion (cyan), Selenium atom (orange), Sulfur (yellow), carbon (dark grey), oxygen (red), nitrogen (blue) and hydrogen (white) atoms are shown as spheres.

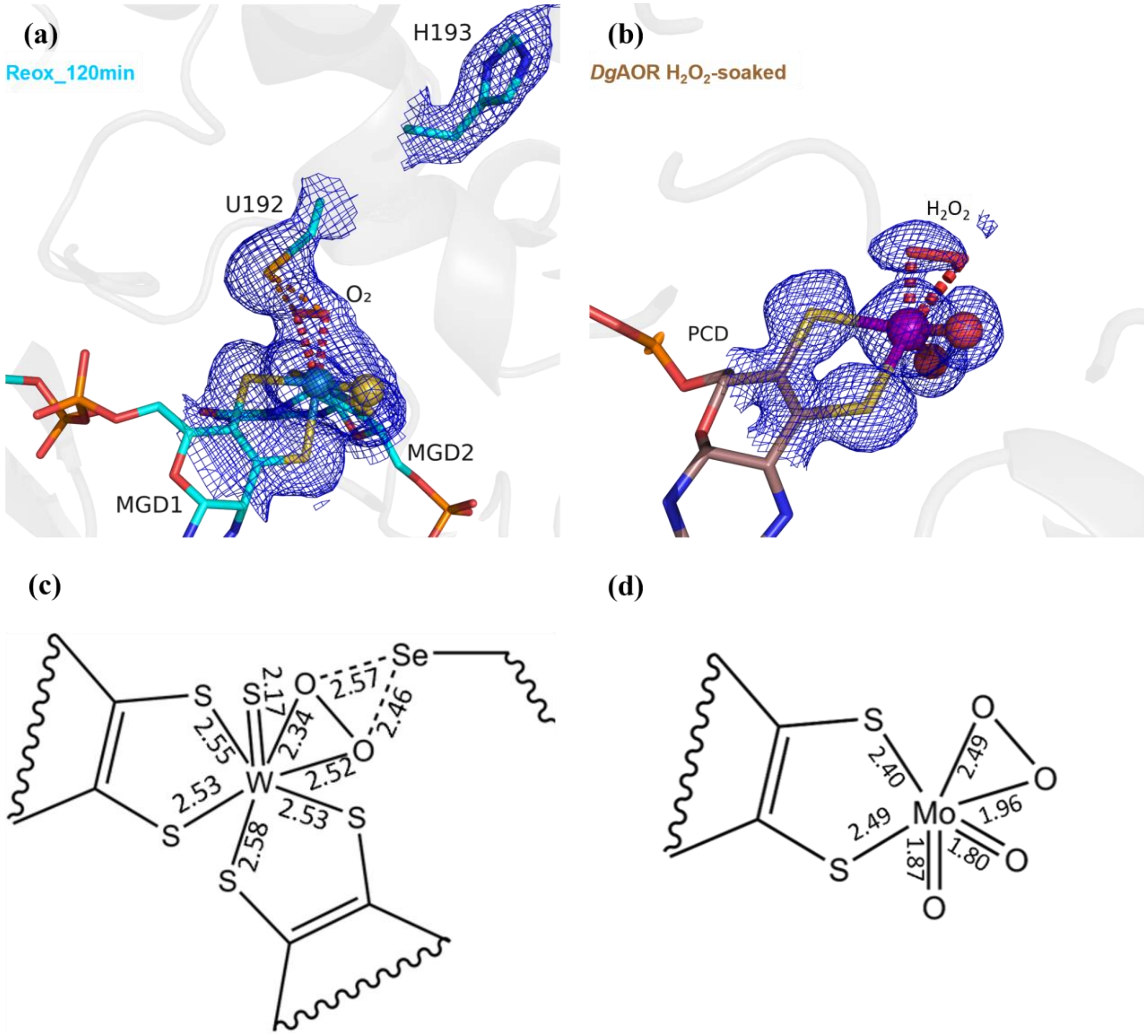

Figure S 7- Comparison of the metal coordination between the dioxygen/peroxide containing active sites of *Dv*FdhAB Reox_120min (cyan) and *Dg*AOR H2O2-soaked (PDB_ID: 4C80) (brown).
(a) *Dv*FdhAB Reox_120min structure (cyan) and respective electron density map (2Fo-Fc), at 1σ (blue mesh). U192, H193, the two MGD co-factors coordinating the W ion and the dioxygen molecule (red) are shown as sticks. (b) *Dg*AOR H2O2-soaked (PDB_ID: 4C80) structure (brown) and respective electron density map (2Fo-Fc), at 1σ (blue mesh). The Molybdopterin Cytosine Dinucleotide (MCD) co-factor coordinating the Mo ion, the two terminal oxo ligands and peroxide molecule (red) are shown as sticks. (c) 2D representation of the *Dv*FdhAB Reox_120min W active site, indicating the bond lengths (in Å) of relevant bonds. (d) 2D representation of the *Dg*AOR H2O2-soaked (PDB_ID: 4C80) Mo active site, indicating the bond lengths (in Å) of relevant bonds.

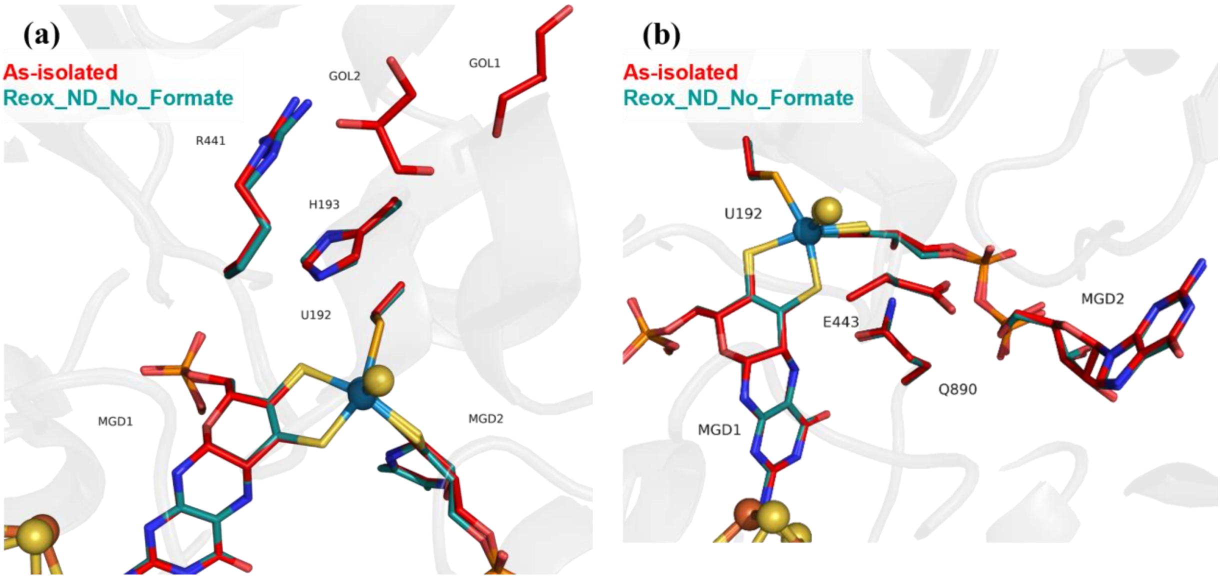

Figure S 8- Superposition of *Dv*FdhAB WT as-isolated (oxidized) (PDB_ID: 6SDR) (red) and oxygen exposed Reox_ND_NoFormate structure (blue) in two different views.
Rmsd is 0.15 Å for 963 Cα atoms of *Dv*FdhA, and of 0.15 Å for 214 Cα atoms of *Dv*FdhB. (a) U192, H193, R441, the two MGD co-factors and two glycerol molecules are shown as sticks. (b) U192, E443, Q890 and the two MGD co-factors are shown as sticks.

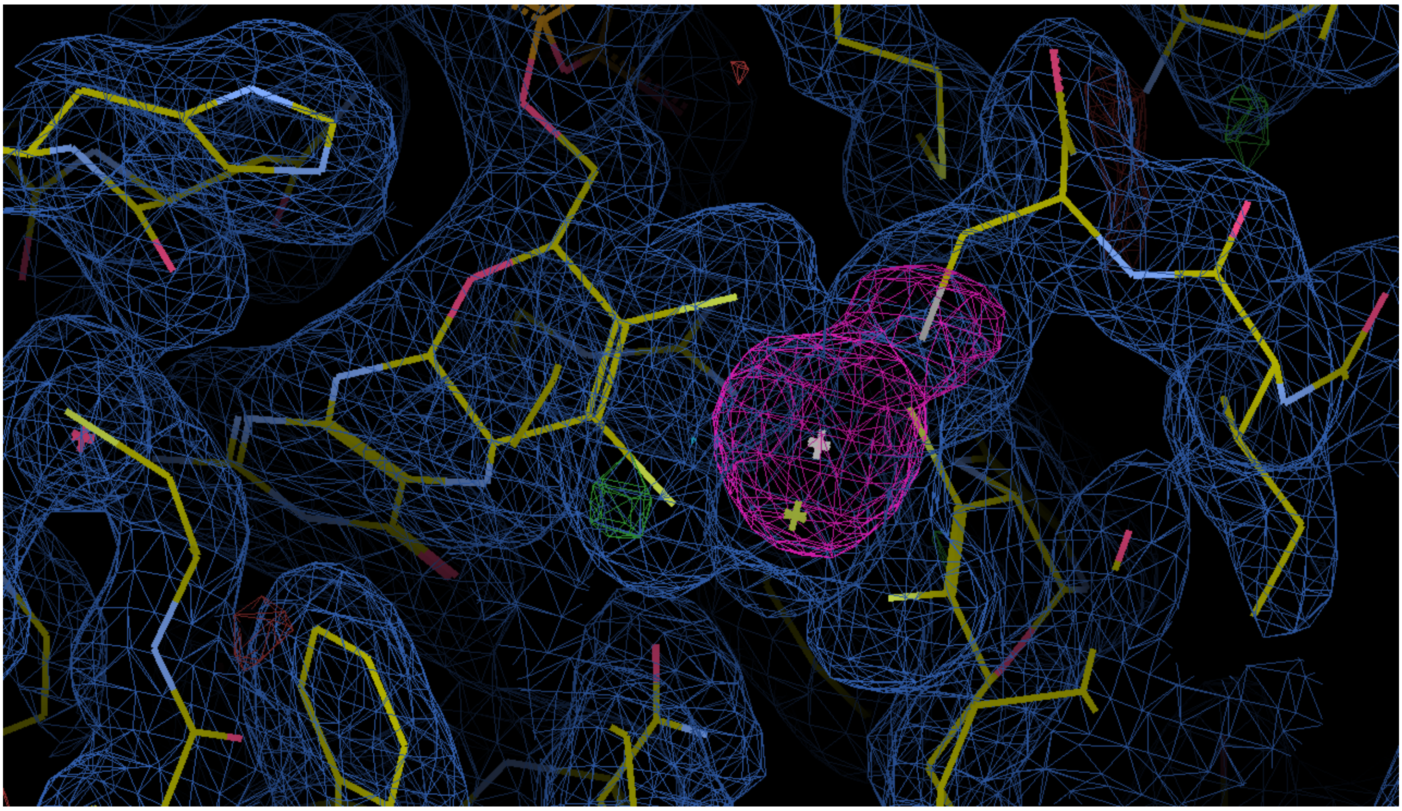

Figure S 9- Control HP experiment with anaerobic *Dv*FdhAB crystals under CO_2 pressure.
Anaerobic, under CO_2 pressure, *Dv*FdhAB structure. The W ion (grey cross), SeCys Se atom (grey line), the two MGD co-factors (yellow lines) and sulfido ligand (yellow cross) are shown. 2Fo-Fc electron density map contoured at 1σ (blue mesh), Fo-Fc map contoured at 3σ (green and red mesh, respectively for positive and negative density) and anomalous electron density map at 3 σ (violet mesh) are shown. Image produced with Coot [4].

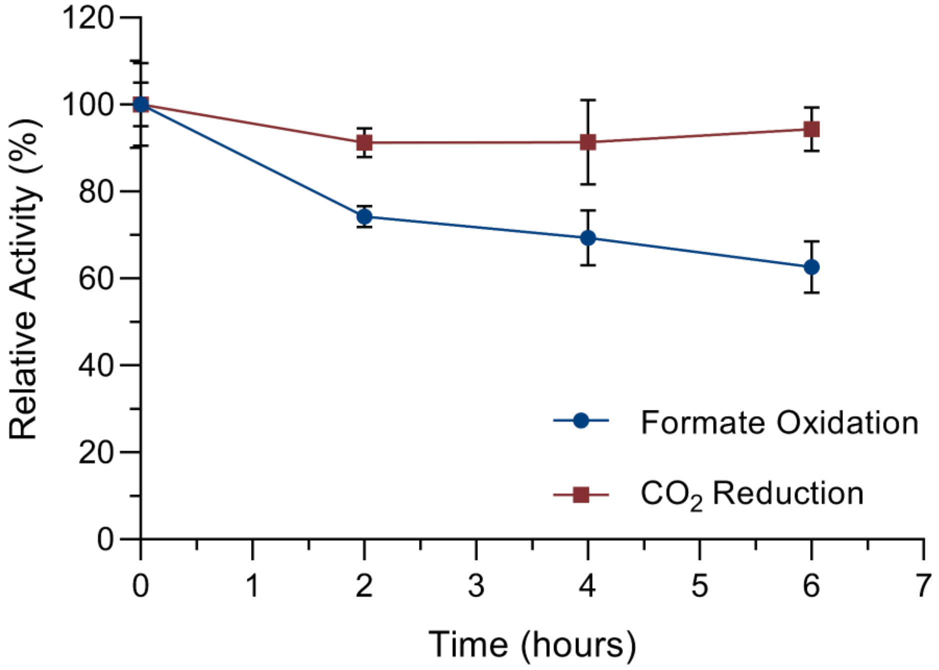

Figure S 10- Relative activity for CO_2 reduction (red) and formate oxidation (blue) of as-isolated *Dv*FdhAB incubated in anaerobic conditions in the presence of CO_2.
T=0h was considered as 100% of activity. Data are presented as mean values ± s.d. (n = 3 assay technical replicates).

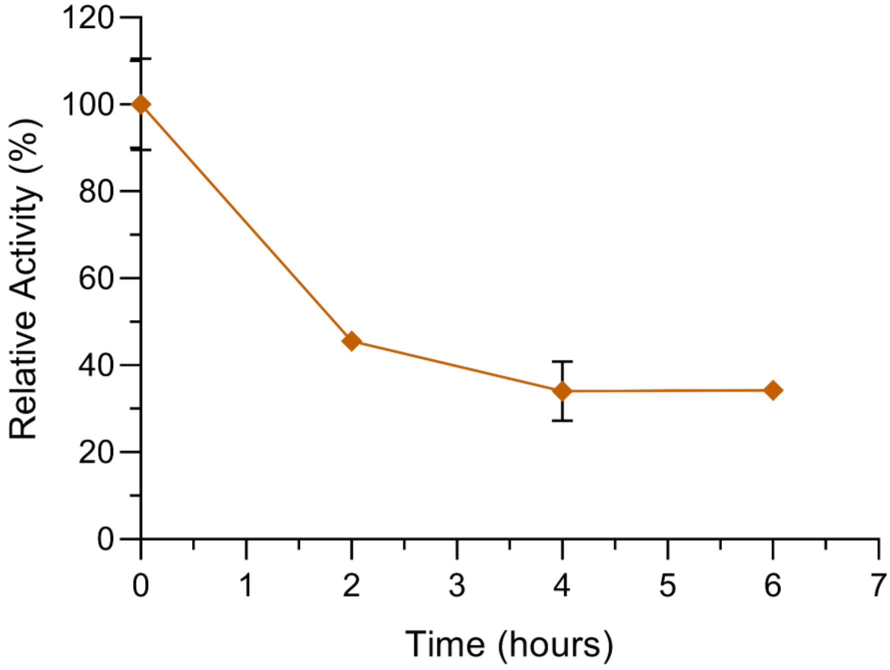

Figure S 11- Relative activity for CO_2 reduction of as-isolated *Dv*FdhAB incubated in aerobic conditions in the presence of CO_2.

T=0h was considered as 100% of activity. Data are presented as mean values ± s.d. (n = 3 assay technical replicates).

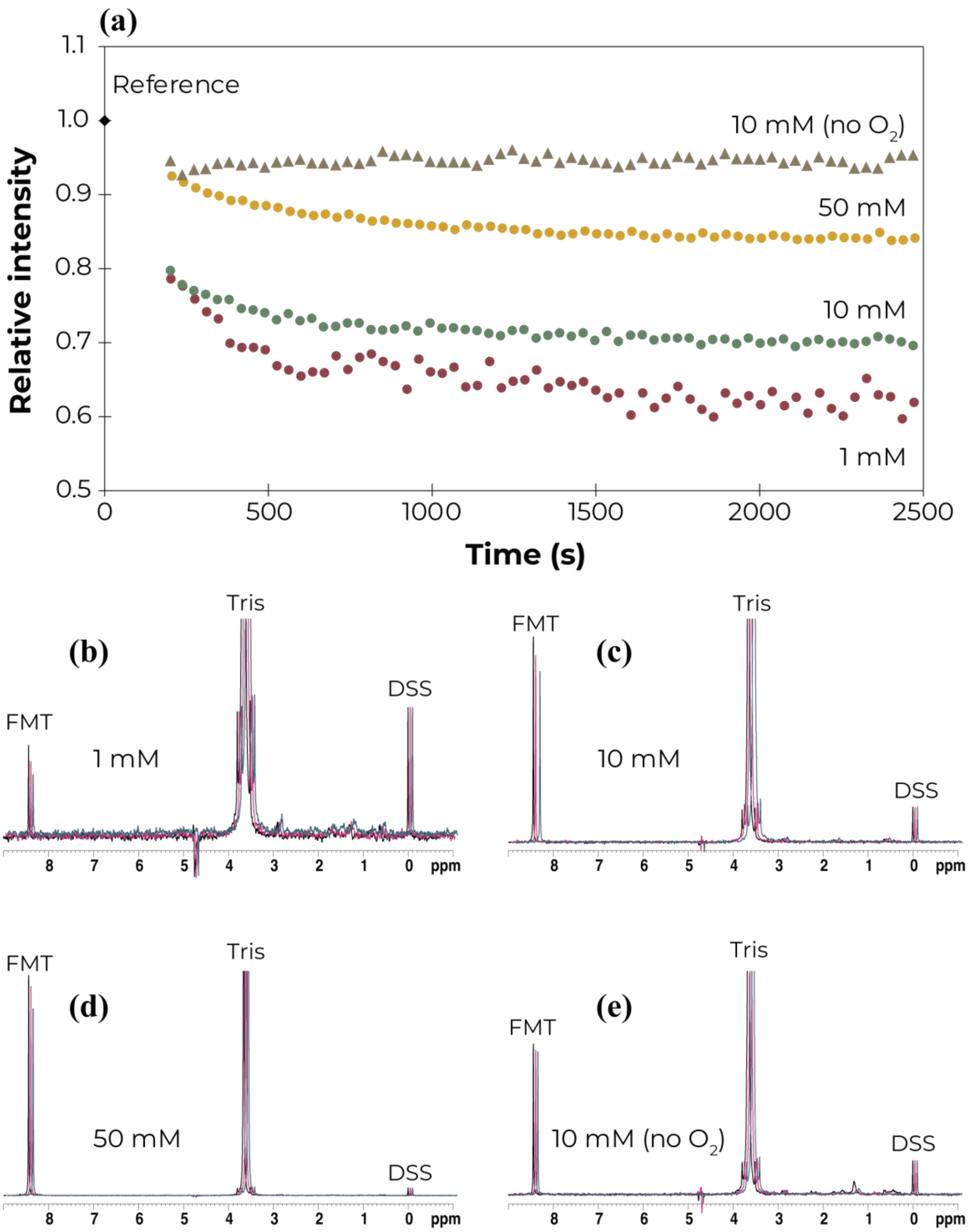

Figure S 12- *Dv*FdhAB activity assays by 1H NMR.
(a) The relative intensity of the 1H NMR formate peak (calculated as the intensity ratio between the formate peak and that of DSS) is plotted as a function of time for the activity assays with formate concentrations of 1 mM (red circle), 10 mM (green circle), 50 mM (yellow circle); and 10 mM in the absence of atmospheric oxygen (grey triangle), the reference (which is calculated in the same way as before, but using the samples prepared in the absence of enzyme) is shown (black diamond). (b - e) Representative 1D 1H spectra of each condition tested (1, 10 and 50 mM formate and 10 mM formate in the absence of atmospheric oxygen, respectively) (black: reference 1H spectrum; magenta: 1st 1H spectrum; blue: last 1H spectrum). Above each spectra the peaks of FMT (formate), Tris (buffer) and DSS are indicated. The spectra corresponding to the 1st and last acquisition points are shifted to the right for better analysis.

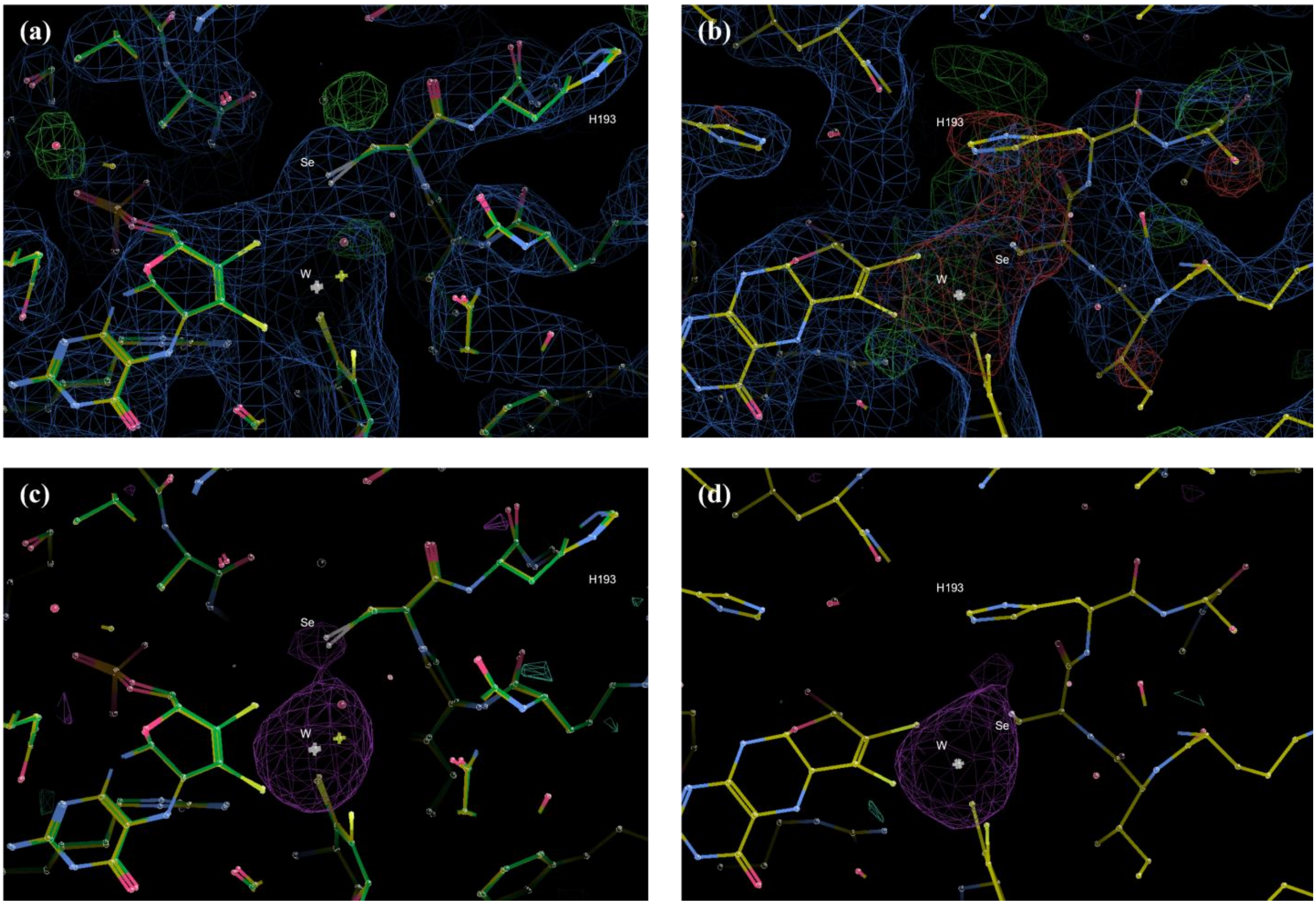

Figure S 13- Solved, unrefined, structure (yellow) obtained from the *Dv*FdhAB WT crystals produced with the concentrated solution left from the NMR experiments with formate under air, at a resolution of 2.83 Å.
2Fo-Fc maps at 1 σ (blue mesh), Fo-Fc maps at 3 σ (green and red mesh, respectively for positive and negative densities) and anomalous map peaks at 3 σ (violet mesh) are shown. Images produced with Coot [4]. (a) and (c) *Dv*FdhAB WT "after-NMR" structure, solved using Reox_120min as molecular replacement model (yellow), superposed with the Reox_120min structure (green). (b) and (d) *Dv*FdhAB WT "after-NMR" structure, solved using *Dv*FdhAB WT as-isolated (oxidized) (PDB_ID: 6SDR) as molecular replacement model (yellow).

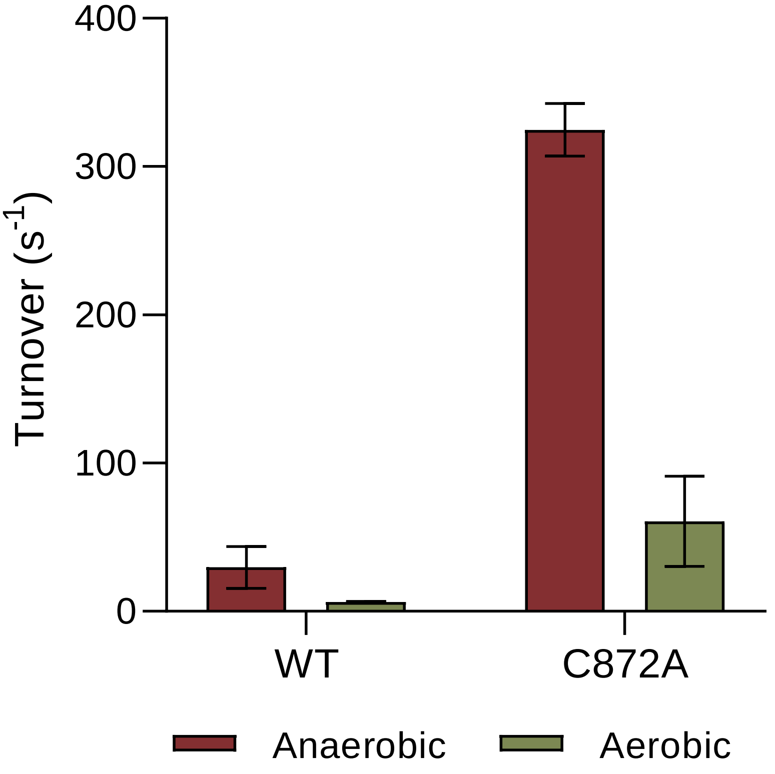

Figure S 14- Formate oxidation activity measured in aerobic and anaerobic conditions, of as-isolated WT *Dv*FdhAB and C872A variant (this variant is equivalent to the DTT-activated form [5]).

The C872A variant corresponds to the active form of FdhAB, equivalent to the form obtained when pretreating the enzyme with DTT, as reported in [5]. With PMS + DCPIP as artificial electron acceptors in anaerobic conditions (dark red, glove box) and in aerobic conditions (green). Data are presented as mean values ± s.d. (n = at least 3 assay technical replicates). No DTT was used in the assays. The higher error observed for the aerobic assay of the C872A variant is due to the decreasing activity of the enzyme in these conditions.

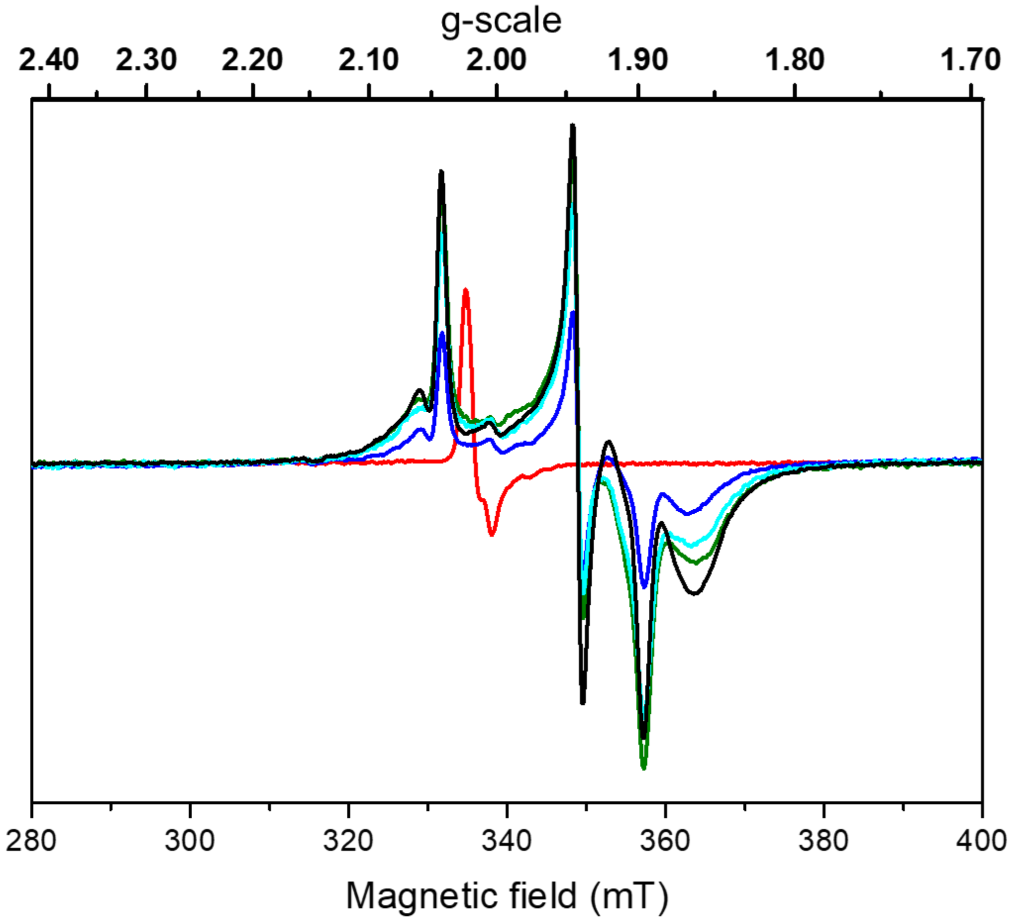

Figure S 15- Influence of formate and oxygen exposure on FeS center EPR signals of *Dv*FdhAB.
Anaerobic reduction with formate (black trace; spin intensity reference, 100%) followed by oxygen treatment (red trace; spin intensity, 2%), then degassing and anaerobic reduction with dithionite (green trace; spin intensity, 105%) or formate (blue trace; spin intensity, 55%), and subsequent reduction of formate treated sample with dithionite (cyan trace; spin intensity, 87%). EPR conditions: Temperature, 15 K; microwave power 1 mW at 9.479 GHz, modulation amplitude 1 mT at 100 kHz.

References

(1) Oliveira, A. R.; Mota, C.; Klymanska, K.; Biaso, F.; Romao, M. J.; Guigliarelli, B.; Pereira, I. C. Spectroscopic and Structural Characterization of Reduced Desulfovibrio Vulgaris Hildenborough W-FdhAB Reveals Stable Metal Coordination during Catalysis. *ACS Chem Biol* **2022**, *17* (7), 1901–1909. https://doi.org/10.1021/acschembio.2c00336.

(2) Oliveira, A. R.; Mota, C.; Mourato, C.; Domingos, R. M.; Santos, M. F. A.; Gesto, D.; Guigliarelli, B.; Santos-Silva, T.; Romão, M. J.; Cardoso Pereira, I. A. Toward the Mechanistic Understanding of Enzymatic CO2 Reduction. *ACS Catal* **2020**, *10* (6), 3844–3856. https://doi.org/10.1021/acscatal.0c00086.

(3) Vilela-Alves, G.; Manuel, R. R.; Oliveira, A. R.; Pereira, I. C.; Romão, M. J.; Mota, C. Tracking W-Formate Dehydrogenase Structural Changes During Catalysis and Enzyme Reoxidation. *Int J Mol Sci* **2023**, *24* (1). https://doi.org/10.3390/ijms24010476.

(4) Emsley, P.; Lohkamp, B.; Scott, W. G.; Cowtan, K. Features and Development of Coot. *Acta Crystallogr D Biol Crystallogr* **2010**, *66* (4), 486–501. https://doi.org/10.1107/S0907444910007493.

(5) Oliveira, A. R.; Mota, C.; Vilela-Alves, G.; Manuel, R. R.; Pedrosa, N.; Fourmond, V.; Klymanska, K.; Léger, C.; Guigliarelli, B.; Romão, M. J.; Cardoso Pereira, I. A. An Allosteric Redox Switch Involved in Oxygen Protection in a CO2 Reductase. *Nat Chem Biol* **2024**, No. 20, 111–119. https://doi.org/10.1038/s41589-023-01484-2.
